# Health-care leaders’ experiences of the competencies required for crisis management during COVID-19: a systematic review of qualitative studies

**DOI:** 10.1108/LHS-10-2022-0104

**Published:** 2023-05-11

**Authors:** Miro Ahti, Leonie Taipale-Walsh, Suvi Kuha, Outi Kanste

**Affiliations:** Research Unit of Health Sciences and Technology, Faculty of Medicine, University of Oulu, Oulu, Finland; Research Unit of Health Sciences and Technology, Faculty of Medicine, University of Oulu, Oulu, Finland and Finnish Centre for Evidence-Based Health Care, Helsinki, Finland

**Keywords:** Competencies, Crisis, Experience, Health care, Leaders, Management, Systematic review, Qualitative, COVID-19

## Abstract

**Purpose:**

This paper aims to synthesize health-care leaders’ experiences of the competencies required for crisis management.

**Design/methodology/approach:**

The systematic review followed the joanna briggs institute (JBI) guidance for systematic reviews of qualitative evidence. The search strategy included free text words and medical subject headings and peer-reviewed qualitative studies published in English, Finnish and Swedish and was not limited by year or country of publication. The databases searched in March 2022 were Scopus, PubMed, CINAHL, ABI/INFORM and the Finnish database Medic. Gray literature was searched using MedNar and EBSCO Open Dissertations. Studies were screened by title and abstract (*n* = 9,014) and full text (*n* = 43), and their quality was assessed by two independent reviewers. Eight studies were included. The data was analyzed using meta-aggregation.

**Findings:**

Fifty-one findings (themes and subthemes) were extracted, and 11 categories were created based on their similarities. Five synthesized findings were developed: the competence to comprehend the operational environment; the competence to stay resilient amidst change; the competence to adapt to and manage change; the competence to manage and take care of staff; and the competence to co-operate and communicate with diverse stakeholders.

**Originality/value:**

This systematic review produced novel information about health-care leaders’ experiences of the competencies required for crisis management during COVID-19. This study complements the field of research into crisis management in health care by introducing five original and unique competency clusters required for crisis management during the acute phase of COVID-19.

## Introduction

Crises differ, and levels of preparedness for crisis management vary nationally and internationally. Crisis can be defined as a major and unpredictable event or emergency situation that threatens to harm individuals and corporations, create instability within organizations and sometimes affect the whole of society (Alvintzi and Eder, 2010). Over the past few years, there has been a significant focus on health crises, following the COVID-19 pandemic which is still causing turmoil in health care. Although the focus has mainly been on COVID-19, it is important to also consider other types of crisis which have an impact on health care, and the competencies involved in managing such crises. Both natural and human-induced disasters can have a huge impact on health care, all over the world. Natural and manmade disasters are global problems that vastly affect health care and its capacity. COVID-19 created a unique opportunity to see how health-care systems and providers responded to a rapidly evolving crisis (Cariaso-Sugay *et al.*, 2021). COVID-19 challenged health-care management and leaders in new and unpredictable ways. A major factor for successfully managing pandemics is having competence in management, where communication, leadership and decision-making proved as particularly important aspects to supporting staff performance and managing this crisis (Jankelová *et al.*, 2021).

During a crisis, the role of leaders is highlighted as requiring particular competence. Competence can be understood as an individual’s capacity to perform and succeed at a given task, while competency describes a specific skill or ability (Seiler and Pfister, 2009). In a professional context, competencies can be described as the abilities that an individual needs and uses in his or her profession (Kapucu and Ustun, 2018). According to Boyatzis (2008), competencies can be understood as a behavioral perspective to emotional, social and cognitive intelligence, and therefore as personal talents which can be developed in time. Consequently, competence development can be considered essential to thrive and excel professionally, notably in the management and leadership positions (Boyatzis, 2009). This review treats competence as an umbrella term. Competence includes knowledge, skills, attitudes (Gunawan and Aungsuroch, 2017), values, capacities and capabilities (Kapucu and Ustun, 2018). Competencies in health-care management can be divided into various domains, as seen in the competency model frameworks published by the American Organization of Nurse Executives (AONE) (2015) and the International Hospital Federation (IHF) (2015). Both frameworks consist of five converging domains of competence:

1. Leadership;
2. communication and relationship management;
3. professionalism, professional and social responsibility;
4. knowledge of health and health-care environment; and
5. business skills and principles. (AONE, 2015; IHF, 2015)

Education and experience can contribute to improving management skills and competencies (Kapucu and Ustun, 2018). Whether competencies are examined solely from the specific perspective of nursing or from the broader perspective of health-care management in general, these domains remain relevant.

Management entails a power dimension, as individuals are entrusted with the leadership of an organization, company, division or team of employees. There is also an ethical dimension to management (Koreimann, 2015). According to Wooten and James (2008), leadership involves different competencies at different stages of managing a crisis, and Van Wart and Kapucu (2011) present that crisis management can provide selective adaptations that cater to the unique circumstances surrounding crisis. Crisis management is generally seen to rest on a few basic principles – prevention, preparedness, response and recovery – of which prevention and preparedness are associated with the pre-crisis phase; response with the crisis phase itself; and recovery with the post-crisis phase (Alvintzi and Eder, 2010). Crisis management can be seen from two primary perspectives: an internal perspective which concentrates on an organization’s in-house operations, and an external perspective which concentrates on the interactions between organizations and their external stakeholders. The internal perspective is concerned with how organizational structures and systems are coordinated to understand, minimize or prevent a crises, while the external perspective considers how outward coordination and communication can help organizations to prevent, solve and move on from crises (Bundy *et al.*, 2017). During a crisis, leaders are responsible for simultaneously managing numerous co-dependent tasks relating to personnel, organizational learning and knowledge management (Buhagiar and Anand, 2021).

During the past few years, several studies of crisis management have been conducted, but our preliminary enquiries suggested that the focus of these studies has been on COVID-19 and the context of an acute crisis phase. Based on a preliminary search of PROSPERO, MEDLINE, the Cochrane Database of Systematic Reviews and JBI Evidence Synthesis, there were no current or in-progress systematic reviews covering health-care leaders’ experiences of the competencies required for crisis management. A systematic review conducted by Ripoll Gallardo *et al.* (2015) focused on core competencies for disaster management and humanitarian assistance and concluded that there is a lack of agreement on the terminology for defining competency. Earlier research into crisis management has focused on particular perspectives including communication and social media, leadership, knowledge, governance, information technology, strategic planning and professional entities (Hazaa *et al.*, 2021). A scoping review by Sriharan *et al.* (2022) focused on crisis leadership during pandemics and concluded that, in such contexts, public health-care leaders draw on task, people and adaptive competencies, which are themselves influenced by political, structural and cultural contexts.

Although health-care leaders’ competence in crisis management has been studied, it seems that the focus has been limited to particular areas of health care, meaning that the literature is fragmented. It lacks a comprehensive systematic review of studies which considers the competencies required for crisis management throughout the field of health care more broadly. We argue that a deeper understanding of the competencies required for crisis management is needed, and that the research topic matters because crises can highlight grievances and reveal shortcomings in operations or management.

The purpose of this review was to synthesize health-care leaders’ experiences of the competencies required for crisis management. The objective was to reveal a deeper understanding of these experiences in a range of health-care settings. The research question was as follows:

RQ1. What are health-care leaders’ experiences of the competencies required for crisis management?

## Research methods

### Study design

This systematic review of qualitative studies considered the experiences of leaders working in health care. The rationale for the study design was based on the link between the JBI model of evidence-based health care and qualitative research study evidence (Pearson *et al.*, 2005), and also between the information need, paradigm and method of synthesis (Korhonen *et al.*, 2013). The need for information on experiences, the purpose to synthesize and the objective to understand those experiences, led to the qualitative paradigm, which in turn led to the process of meta-synthesis using meta-aggregation.

### Data sources and search strategies

The systematic review followed the JBI guidance for systematic reviews of qualitative evidence (Lockwood *et al.*, 2020). The search strategy aimed to identify both published and unpublished studies. An initial limited search of Scopus, PubMed, CINAHL and the Finnish database Medic was done to identify relevant articles. Specific terms contained in the titles, abstracts and index descriptions (i.e. MeSH) of relevant articles were used to develop a full search strategy (Supplementary Table 2). This search strategy was developed in cooperation with an information specialist.

The search strategy, including all identified keywords and index terms, was adapted for each information source included. Only peer-reviewed studies and research articles published in English, Finnish and Swedish were included. The search strategy was not limited by year or country of publication. The databases searched on March 13, 2022, were Scopus, PubMed, CINAHL, ABI/INFORM and the Finnish database, Medic. MedNar and EBSCO Open Dissertations were searched for relevant grey literature. The reference lists from all studies selected for critical appraisal were screened for any additional relevant studies.

### Study selection

The inclusion and exclusion criteria were developed using PICo: (P) participants, (I) phenomena of interest, (Co) context (Table 1).

Following the search, all identified citations were collated and uploaded into the bibliographic management system Covidence (version 2.0) and duplicates were removed. The titles and abstracts were screened by two independent reviewers (M.A. and L.T-W.) for assessment against the inclusion criteria. The full texts of selected citations were then assessed in detail against the inclusion criteria by the same two independent reviewers (M.A. and L.T-W.). Only empirical studies, where quotations of respondents were available and indicated by a unique participant ID, were included to recognize how diversely the original researchers used participants’ verbatim quotations. Reasons for exclusion were recorded (Supplementary Table 3). Disagreements between the reviewers were resolved through discussion, and the eligibility of three studies was discussed further with a third reviewer (O.K.).

**Figure 1.**
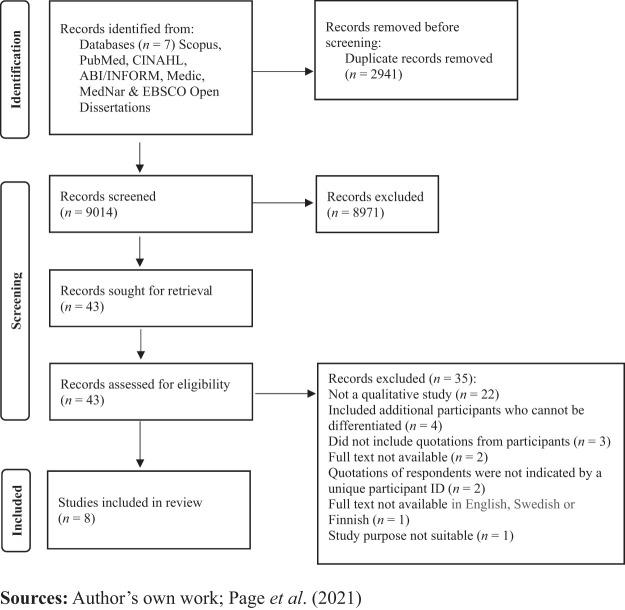
Illustration of the search process, including inclusion criteria and study selection (PRISMA 2020 statement)

The results of the search and the process for inclusion in the study are reported in full and presented in a Preferred Reporting Items for Systematic Reviews and Meta-analyses (PRISMA) flow diagram (Page *et al.*, 2021) (Figure 1). The database search identified 9,014 studies after removing duplicates. Having excluded 8,971 articles based on their titles and abstracts, the full texts of 43 articles were reviewed. Of these, 35 were excluded because they did not meet the inclusion criteria, leaving eight studies for critical appraisal. The reference lists from all of the studies selected for critical appraisal were screened for additional sources. No further studies were identified through manual searching. Eight qualitative studies were included.

### Quality assessment

Eligible studies were critically appraised for methodological quality by two independent reviewers (M.A. and L.T-W.) using the JBI critical appraisal checklist for qualitative research (Lockwood *et al.*, 2015) (Supplementary Table 4). Disagreements between the reviewers were resolved through discussion. Following the appraisal, studies that would not have met a quality threshold of at least five points out of ten would have been excluded, after considering which question on the checklist has not been met with an affirmative “yes.” All eight included studies met the quality threshold.

### Data extraction and synthesis

Data was extracted by two independent reviewers (M.A. and L.T-W.). The data extraction included citations, purpose, participants, methodology, key findings and critical appraisal (Supplementary Table 1). Only findings relevant to the review’s research question were extracted. Findings were identified from themes and subthemes within the results sections of the studies included. Illustrations supporting these findings, such as direct quotations in the participants’ voice were then extracted, and the findings, with their accompanying illustrations, grouped to generate categories based on similarities in their content. Categories and synthesized findings, with their respective accompanying descriptions, were created and finalized through a consensus process involving discussion between the two independent reviewers (M.A. and L.T-W.).

The data was analyzed using a qualitative meta-aggregation approach developed by the JBI (Lockwood *et al.*, 2015), which aims to synthesize qualitative evidence (Lockwood *et al.*, 2020). The findings (themes and subthemes) were extracted and assessed against the illustrations provided (verbatim quotes from research participants) in the studies selected. Levels of credibility were assessed based on how the findings and the illustrations provided corresponded with each other. Findings were then classified according to the following levels (Supplementary Table 5): unequivocal (U) findings are accompanied by an illustration that is beyond reasonable doubt and therefore not open to challenge; credible (C) findings are accompanied by an illustration that lacks clear association with that finding and therefore is open to challenge; and not supported (NS) findings are not supported by the data (Lockwood *et al.*, 2015; Munn *et al.*, 2014). Findings that were not supported, and therefore not included in the synthesis, are illustrated in Supplementary Table 6.

The ConQual approach was used to establish confidence in the evidence, in accordance with the JBI’s guidelines for systematic reviews of qualitative evidence. The summary of findings (Supplementary Table 7) includes the ConQual score calculated for each synthesized finding. This score is based on the strength of the evidence that informs the relevant findings. Based on measures of dependability and credibility of the included studies, the overall ConQual score may be high, moderate, low or very low. (Munn *et al.*, 2014).

## Findings

### Overview of the reviewed studies

The qualitative studies included (n = 8) were conducted in Australia (*n* = 1), Canada (*n* = 1), Denmark (*n* = 1), Jordan (*n* = 1), Spain (*n* = 1), the UK (*n* = 1) and the USA (*n* = 2). The year of publication ranged from 2021 to 2022. Most of the studies were conducted in hospital settings. The number of participants ranged from 6 to 16. Participants were mainly leaders from nursing professions (Supplementary Table 1). In this review, all studies met the critical appraisal score of eight points out of ten (Supplementary Table 4). All of the studies included focused on the COVID-19 crisis.

In total, 51 findings were extracted, and 11 categories were generated and grouped into the following five synthesized findings:

1. the competence to comprehend the operational environment;
2. the competence to stay resilient amidst change;
3. the competence to adapt to and manage change;
4. the competence to manage and take care of staff; and
5. the competence to co-operate and communicate with diverse stakeholders (Supplementary Table 8, Supplementary Figure 1).

The overall ConQual score calculated for each synthesized finding was low (Supplementary Table 7). Because the levels of credibility and dependability were moderate, and the ConQual scores low, the evidential value of the results should be regarded as merely suggestive.

### Competence to comprehend operational environment

The first synthesized finding, which was based on two categories of findings, shows that the contradiction between the internal and external operational environment requires skills in understanding and acknowledging the varying needs of different stakeholders, while simultaneously ensuring patient-oriented care.

The first category, “Ensuring patient-oriented care,” included two findings: patients’ transitions through the health-care system (Jackson and Nowell, 2021); and preservation of humanized care (Vázquez-Calatayud *et al.*, 2022). One leader’s experience of patients’ transitions through the health-care system focused on maintaining the fluency of the care pathway: “How do we get them out of this unit safely to the front doors […] (M7).” (Jackson and Nowell, 2021, p. 2396), while another’s experience centered on the preservation of humanized care: “Do not forget about the person. […] we have not been able to care as perhaps we would have liked to care […] (NM10)” (Vázquez-Calatayud *et al.*, 2022, p. 85).

The second category, “Balancing the various needs of stakeholders,” included four findings: staff needs versus overall needs; loyalty to own leaders versus staff need; upholding guidelines versus relatives’ and patients’ needs (Hølge-Hazelton *et al.*, 2021); and navigating the political climate (Jackson and Nowell, 2021). One leader’s experience of the latter focused on balancing the quality of care with occupational safety: “ […] The patients should be treated well, but we also needed to protect ourselves. (WM-A)” (Hølge-Hazelton *et al.*, 2021, p. 1407).

### Competence to stay resilient amid change

The second synthesized finding shows that resilience requires the ability to lead during uncertainty, cope on a personal and professional level and use skills that one has already acquired through formal education and reflection. Two categories contributed to this synthesized finding.

The first category, “Learning and transitioning through change,” included eight findings: practicing distance leadership; being on a steep learning curve; management education provides valuable tools; personal development - rewound (Hølge-Hazelton *et al.*, 2021); changing roles and responsibilities (Jackson and Nowell, 2021); reflections on learning; personal coping (White, 2021); and self-awareness (Abu Mansour and Abu Shosha, 2022). Personal development - rewound was showcased by one leader’s experience of reflecting on what they had learned about their own leadership in the midst of a crisis: “On a personal level this (pandemic) has given much, I have never tried something like this before […] (WM-E)” (Hølge-Hazelton *et al.*, 2021, p. 1408).

The second category, “Enduring uncertainty throughout change,” included four findings: managing uncertainty (Vázquez-Calatayud *et al.*, 2022); planning during uncertainty (Jackson and Nowell, 2021); extraordinarily demanding system; and physically demanding situations (Abu Mansour and Abu Shosha, 2022). One leader’s experience of managing uncertainty was that one should always be prepared for crisis situations ahead of time: *“*Each day you came to work, it was something different […] the most important thing was to get ahead of events, […] that they never caught us unaware. (NM4)” (Vázquez-Calatayud *et al.*, 2022, p. 84).

### Competence to adapt to and manage change

The third synthesized finding suggests that adaptability in managing change requires the skills to find new solutions for dealing with imminent challenges and emerging situations. It draws on two categories of findings.

The first category, “Embracing new and flexible ways of working,” included eight findings: flexible work approach and practices; expanded ways of working; removal of organizational barriers (Riddell *et al.*, 2022); urgent and constant reorganization of the service (Vázquez-Calatayud *et al.*, 2022); compensating for shortage of materials and human resources (Abu Mansour and Abu Shosha, 2022); innovation, leadership, management and planning (Roche *et al.*, 2021); and revamping my approach (White, 2021). One leader’s experience focused on innovation, being open and receptive to this and the benefits it provided: “Brilliant changes with all the online stuff, webcam stuff, future world opportunities- less travel, environment, time benefits, safety. (pt 4)” (Roche *et al.*, 2021, p. 4), while another leader’s experience of the urgent and constant reorganization of a service involved constantly looking for new and alternative solutions: “[…] We had to search for alternatives to the problems that came up and solve them quickly […] (NM6)” (Vázquez-Calatayud *et al.*, 2022, p. 83).

The second category, “Coping with changing resources,” included four findings: complexity of staff management in a changing situation (Vázquez-Calatayud *et al.*, 2022); designating and transferring staff (Hølge-Hazelton *et al.*, 2021); workplace transitions in response to COVID-19; and maintaining quality through problem-solving (Jackson and Nowell, 2021). One leader’s experience demonstrated the need to be able to be professional rather than idealistic when making decisions about allocating and transferring staff: “My own values have been overruled. […] I had to use my professional side not my emotional side when deciding whom to transfer […] (WM-C)” (Hølge-Hazelton *et al.*, 2021, p. 1405).

### Competence to manage and take care of staff

The fourth synthesized finding suggests that managing and supporting staff requires the skill to make best use of staff members’ knowledge and build their expertise in relevant topics as needed. Three categories of findings contributed to this synthesized finding.

The first category, “Maintaining an active grip and presence in leadership,” included nine findings: having a leadership presence (Losty and Bailey, 2021); reliance on me (White, 2021); maintaining presence, own leadership virtues and professionalism; bottom-up decision-making (Hølge-Hazelton *et al.*, 2021); participation in decision-making (Vázquez-Calatayud *et al.*, 2022); expanded working relationships; knowledge development and dissemination (Riddell *et al.*, 2022); staff development; and maturity of management skills (Abu Mansour and Abu Shosha, 2022). One leader’s experience highlighted the issue of reliance the leader and the need to be available to staff at all times: “My job was 24/7. […] I know the staff needed me. If I wasn’t there, I worried that they would not have what they needed and know about the new directives […] (NM)” (White, 2021, p. 1530). Another leader’s experience related to staff development, communicating with staff and growing their situational awareness: “We have been trained with the new equipment and are constantly provided with new issues related to the disease after receiving sufficient information and comprehensive instructions. […] we communicated with the staff to convey information and enhance their awareness […] (Participant 3)” (Abu Mansour and Abu Shosha, 2022, p. 388). Another experience shared by a leader related to knowledge development and dissemination, specifically being able to convey one’s knowledge and take an active lead when needed: “ […] one of the things I’m really incredibly proud of is the training team that [we] stood up virtually overnight […] (Participant 014)” (Riddell *et al.*, 2022, p. 9).

The second category, “Understanding the capability of the workforce,” included two findings: workforce development and training (Roche *et al.*, 2021); and mental toughness (Losty and Bailey, 2021). One leader’s experience of mental toughness involved recognizing staff excellence during difficult situations: “ […] it was amazing how innovative and flexible the nurses became when faced with uncertainty. (NE01)” (Losty and Bailey, 2021, p. 121).

The third category, “Taking care of the well-being of staff,” included three findings: prioritization of the biopsychosocial well-being of staff (Vázquez-Calatayud *et al.*, 2022); work that needs attention going forward; and a different kind of support (White, 2021). One leader’s experience related to prioritizing the biopsychosocial well-being of staff: “My priority […] was to make sure they didn’t lack anything […] They were calmer when they talked and said what they thought. (NM2)” (Vázquez-Calatayud *et al.*, 2022, p. 85).

### Competence to co-operate and communicate with diverse stakeholders

The fifth synthesized finding indicates that co-operation and communication requires skills for multidisciplinary collaboration, being aware of the situation at hand and being able to convey the necessary information to all parties concerned. Two categories fed into this synthesized finding.

The first category, “Ensuring effective communication,” included three findings: communication is paramount (Losty and Bailey, 2021); extensive information and communication (Riddell *et al.*, 2022); and communication (Roche *et al.*, 2021). One leader’s experience related to communication concerned the distribution of knowledge across all organizational levels: “There were daily management meetings where information dissemination from the top and clinicians relaying frontline experience. (pt 6)” (Roche *et al.*, 2021, p. 4).

The second category, “Sustaining teamwork and collaboration,” included four findings: teamwork; collaboration (Vázquez-Calatayud *et al.*, 2022); professional support (White, 2021); and colleagues’ support (Abu Mansour and Abu Shosha, 2022). One leader’s experience highlighted colleagues’ support: “We all support each other and work as a team […] (Participant 1)” (Abu Mansour and Abu Shosha, 2022, p. 389).

## Discussion

This systematic review produced novel information about health-care leaders’ experiences of the competencies required for crisis management during COVID-19. This study complements the field of research into crisis management in health care by introducing synthesized key findings i.e. five original and unique competency clusters, required for crisis management during the acute phase of COVID-19. Arguably, as such, these kind of competency clusters have not been presented in previous research, albeit some of the competencies they consist of have been recognized, which is considered in further detail below.

The first key finding is that health-care leaders must be competent to comprehend the operational environment in question. The contradiction between the internal and external operational environment requires skills in understanding and acknowledging the varying needs of different stakeholders, while simultaneously ensuring patient-oriented care. Our findings showed that health-care leaders’ experiences of the competencies required for crisis management combine the internal and external perspectives of the operational environment, rather than distinguishing between them. Balancing the various needs of stakeholders is an original category of findings in this study that has previously yet to be widely recognized. Our findings suggest that the various needs of different stakeholders affect the operational environment simultaneously, making it incoherent and complex, which requires the competence to recognize this and continually balance between them. Previous studies (Abdi *et al.*, 2022; Bundy *et al.*, 2017) have recognized different stakeholders’ needs. However, their focus is limited to considering them as separate rather than interrelated but also contradictory entities, which is a novel finding in our study. Our findings on ensuring patient-oriented care, i.e. the competence to consider patients’ needs and experiences, focused on patients’ transitions through the health-care system and the preservation of humanized care. Previously, Lyng *et al.* (2021) have examined the topic of patient-centered care from a service innovation perspective, and Ozmen and Arslan Yurumezoglu (2022) in the context of emotional responses in terms of empathy. Our findings contribute to a further understanding multi-stakeholder needs and patient-oriented care and care pathway.

The second key finding is that health-care leaders need the competence to be resilient amidst change, where resilience means being able to lead despite uncertainty, cope on a personal and professional level and use the skills that they have acquired through their education and experience to date. Our findings lean toward professionality as a source of resiliency. Our findings on learning and transitioning through change, i.e. the competence to grow, adapt, reflect and learn from change, show that skills acquired through formal education can be used in managing crises, which is supported by reflection and continuous learning. Previous studies have noted the significance of formal education and training (Abdi *et al.*, 2022; Wooten and James, 2008) and lifelong learning (Stanić *et al.*, 2022). Moreover, leadership training using evidence-based approaches has been shown to affect nurse leaders’ knowledge and confidence in disaster management (Cariaso-Sugay *et al.*, 2021). Previous studies have stated that formal education solely may not provide the particular competencies needed for crisis management in the public sector (Kapucu and Ustun, 2018). Our findings show that the experiences gained from a crisis can help leaders to define what kind of leadership they want to pursue moving forward. Thus, it seems that the practical experience of work and leadership should back up personal and professional competencies acquired through formal education. Our findings show that enduring uncertainty throughout change, i.e. the competence to stay resilient during uncertainty, consists of managing and planning during uncertainty, extraordinarily demanding systems and physically demanding situations. Our findings emphasize managerial, physical and emotional endurance. Previously, the importance of personal coping has been recognized as emotional regulation as the means to cope in times of difficulty (Shih *et al.*, 2009). In comparison, Mackay *et al.* (2022) have presented self-management as part of intrapersonal competencies, which entail setting limits to preserve work–life balance. Our findings differed in depicting endurance without limits and presenting physical endurance as a novel finding.

Our third key finding is that health-care leaders must be able to adapt to and manage change which, in turn, requires that they are skilled at finding new solutions and dealing with imminent challenges and changing situations. This can be framed as leadership, management and decision-making. However, it also involves flexibility and innovativeness on a personal and organizational level and effective management of material and human resources. Previous studies have also regarded adaptive competencies needed amid pandemic situations in terms of decision-making, systems thinking/sense-making and tacit skills (Sriharan *et al.*, 2022). Embracing new and flexible ways of working consists of the competence to tolerate change and respond creatively. Our findings show that leaders embraced innovativeness and innovation in their organization and strived to be creative. Previously innovations and innovativeness have been linked to resilience (Lyng *et al.*, 2021). Innovativeness at the organizational level and creativity at the individual level has previously been presented by Kapucu and Ustun (2018), which is also supported by our findings. Coping with changing resources requires the competence to be resourceful while managing staff through prioritization and allocation. Our findings show that resource management competence is valuable, although the focus was predominantly on human resources and recognizing the value of upholding the quality of care. Previous studies have also considered the value of resource management (Abdi *et al.*, 2022; Stanić *et al.*, 2022) and pragmatic decision-making under severe time and resource constraints (Abdi *et al.*, 2022; Van Wart and Kapucu, 2011). Our findings considered preparedness and planning modestly, although they can be seen as core task-related competencies for crisis management (Sriharan *et al.*, 2022). Our findings emphasized people-centeredness as the key to resource management and contributed by highlighting human resourcing as a whole, over material resourcing.

The fourth key finding is that health-care leaders need the competence to manage and take care of staff, particularly by using staff members’ knowledge and enabling them to develop new, relevant expertise. The category of maintaining an active grip and presence in leadership consists of the competence to support staff, participate in decision-making, share awareness of the situation and maintain open dialog. This can be seen through leaders giving their employees a more active role in decision-making but not relinquishing their leadership responsibilities, which is also necessary for managing emergencies (Van Wart and Kapucu, 2011). Our findings on the competence to support staff echo previous studies. Previous findings have been presented in the context of people competencies regarding presence, empathy and awareness (Sriharan *et al.*, 2022). Furthermore, in the context of leadership and supervisory competencies, in terms of being present and available for the teams (Mackay *et al.*, 2022). The category of understanding the capability of the workforce consists of the competence to identify developmental needs and acknowledge accomplishments. Our findings on workforce development and training are based on situational needs, whereas Abdi *et al.* (2022) have based training needs on understanding the strengths and weaknesses of the employees. Our findings present that leaders recognized the staff’s work in terms of mental toughness. Even though acknowledging accomplishments has previously been considered in theory (Abdi *et al.*, 2022; Dirani *et al.*, 2020), our findings now illustrate it being used in practice. Taking care of the well-being of staff consists of the competence to recognize the individuality of the workforce. This can be seen as supporting staff in ensuring they had supplies and considering their work–life balance when arranging shifts. Previously caring about the well-being of others has been recognized by Sriharan *et al.* (2022), whose findings about people competencies focus on managing interpersonal relationships in the response phase of a pandemic. Furthermore, interpersonal competencies have been presented in terms of listening to teams, discussing more personal matters and distributing the hours worked in the team (Mackay *et al.*, 2022). Abdi *et al.* (2022) have considered well-being and safety in optimizing the workforce and its resources applicable to the situation. Jankelová *et al.* (2021) have presented that leaders can facilitate conditions relating to employee performance in terms of job satisfaction, creating safe working environment and working conditions and support and reduction of stress. Our findings recognized the need to provide and publicize easily accessible programs for stress for employees in the future, which supports the importance of providing psychological support in the workplace (Jankelová *et al.*, 2021). Another example would be to change the mindset of teams in terms of relaxing the atmosphere (Mackay *et al.*, 2022), even though this type of approach was not recognized in our findings.

Our fifth and final key finding is that health-care leaders require the competence to co-operate and communicate with diverse stakeholders. This requires skills for multidisciplinary collaboration, being aware of the situation at hand and being able to convey the necessary information to all parties concerned. Ensuring effective communication requires the competence to handle, receive and distribute information. Our findings show that communication is multidirectional, i.e. inward and outward between organizations, top-down and down-top within the organization and dealing with multiple sources of information. The importance of crisis communication is supported by previous studies where communication can be seen as an essential competency for crisis management (Abdi *et al.*, 2022; Sriharan *et al.*, 2022; Jankelová *et al.*, 2021). During the acute stage of a crisis, the emphasis should be on sharing reliable information quickly (Jankelová *et al.*, 2021). Sustaining teamwork and collaboration involves the competence to collaborate, work within a team and give and receive support. Our findings show that a crisis tightened personnel collaboration within a team, between personnel and leaders and between teams within the organization. Previously, the support of colleagues and other multidisciplinary teams has been seen to ease the work of leaders during the pandemic (Ozmen and Arslan Yurumezoglu, 2022). This can also be seen in our findings. It has been suggested that team performance during the acute crisis stage may be supported by trustworthy, transparent and rapid information sharing (Jankelová *et al.*, 2021). Our findings show that leaders observed team unity contributing largely toward team performance. Previous studies show that networking and partnering are essential means for communicating during a crisis (Abdi *et al.*, 2022), and communicating and co-operating with stakeholders can shape their perspectives on the crisis at hand (Bundy *et al.*, 2017). Our findings emphasize co-operation, collaboration and communication within the organization rather than networking with various stakeholders. Communication with various stakeholders was seen modestly in our findings, and when presented, the focus was on informing the public. Interestingly, health-care leaders focus on operational development within their organization rather than networking and collaborating with other organizations.

The five synthesized key findings, i.e. original and unique competency clusters, illustrate that health-care leaders’ experiences of the competencies required for crisis management during COVID-19 comprise numerous competencies over a wide range of subject areas which enable them to focus on the management of the operational environment, resilience, change, staff and other resources and communication and collaboration. Our findings are also supported by the various competency domains (AONE, 2015; IHF, 2015). Crisis management during COVID-19 requires the competence to manage and lead an organization in both an inward-facing and outward-facing manner. Thus, during a crisis such as COVID-19, leaders should expand their leadership and management competencies to address the diversity and complexity of the situation.

### Limitations

This review was subject to some limitations. Although a systematic approach was used, some relevant studies may not have been included due to the limited use of search terms and languages in the search strategy. The review consists of only eight qualitative studies. All but one of these studies were conducted in western countries, raising questions about their transferability to other contexts. Due to the moderate levels of credibility and dependability of the findings and the low ConQual scores of the synthesized findings, the evidential value of the results should be regarded as merely suggestive. The original aim was to reveal a deeper understanding of health-care leaders’ experiences of the competencies required for crisis management in various health-care settings. The search did not reveal papers that enabled this, so we could only partially achieve the original aim. Most participants were nurse leaders, so the review is mainly limited to their experiences. It cannot be considered to reflect the experiences of all health-care leaders. The context was limited mainly to hospital settings and COVID-19: accordingly, it is not representative of other types of crises in more varied health-care settings. Although the studies included are topical, and no time limit was applied in the search strategy, they represent the narrow timeframe of 2021–2022.

## Conclusion

The review presents five key conclusions and their respective recommendations. The competence to comprehend the operational environment requires skills for understanding and acknowledging the contradiction between internal and external operational environments and stakeholders’ diverse needs while ensuring patient-oriented care. The competence to stay resilient amidst change requires the ability to lead in contexts of uncertainty, cope on a personal and professional level and use skills that leaders have acquired through formal education and reflection. The competence to adapt to and manage change requires skills for finding new solutions to dealing with imminent challenges and dynamic situations. The competence to manage and take care of staff requires the skills to manage and take care of staff, particularly making good use of staff members’ knowledge and enabling them to develop new, relevant expertise. The competence to co-operate and communicate with diverse stakeholders requires skills in multidisciplinary collaboration, awareness of the situation and conveying the necessary information to all parties concerned.

This review complements the field of research into crisis management in health care by introducing synthesized findings from qualitative studies regarding health-care leaders’ experiences of the competencies required for crisis management during COVID-19. This review offers synthesized findings based mainly on nurse leaders’ experiences within the context of COVID-19 and focuses on crisis management in the acute phase of the pandemic. These synthesized findings provide a deeper understanding of crisis management competence and contribute to developing operations and management within health care. On a practical and societal level, the presented synthesized findings can also lead the discussion of whether health-care leaders’ competencies are sufficient to face the next crisis. Further research could consider comparing health-care leaders’ experiences of the competencies required for crisis management during COVID-19 with published crisis management frameworks and models. In addition, further research should consider empirical, quantitative and mixed methods approaches and encompass both pre- and post-crisis phases.

## Figures and Tables

**Table 1.** Inclusion and exclusion criteria and search terms, using PICo

PICo	Inclusion criteria	Exclusion criteria	Search terms
Participants (P)	Health-care leaders	Health-care professionals who were not in a leadership position, not recognized leaders or were health-care students	MH Leader, leader, leaders, manager*, MeSH Term/MH “nurse administrators,” (Medic: johtami*)
Phenomenon of interest (I)	Experiences of competence required for crisis management	Crisis management competency models and frameworks	competen*, knowledge, skill*, attribute, attitude*, expertise, knowhow, capabilit*, capacit*, qualification*, abilit*, MeSH Term/MH “professional competence,” MeSH Term/MH “decision making”
Context (Co)	Various types of crisis in various health-care settings	Crises outside the context of health care	crisis, crises, disaster*, emergenc*, pandemi*, MeSH Term/MH “disease outbreaks,” MeSH Term/MH “natural disasters,” (Medic: kriisi) “health care,” healthcare

**Source:** Authors’ own work

## Supplementary material

The supplementary data for this article can be found online.
