# Supplementary material for: Health-care leaders’ experiences of the competencies required for crisis management during COVID-19: a systematic review of qualitative studies
Source: Leadersh Health Serv (Bradf Engl). 2023 May 11;36(4):595–610. doi: 10.1108/LHS-10-2022-0104 (PMC10853848; doi:10.1108/LHS-10-2022-0104)
Supplement: Supplementary file 1 [file leadershhealthserv-36-0595-s001.docx]

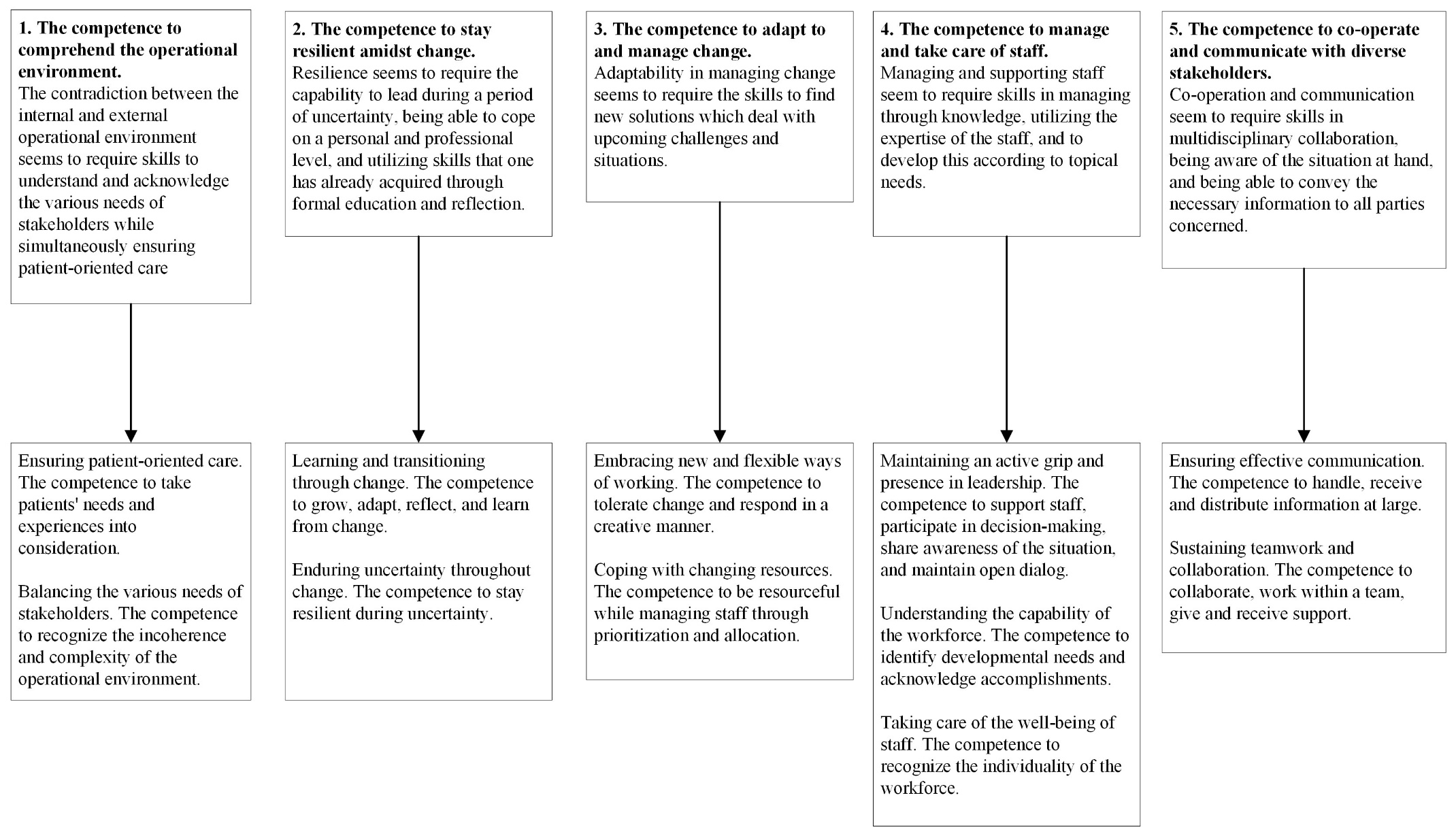


Supplementary Figure 1: Overview of the five synthesized findings, i.e. competency clusters, and their categories (Source: Authors own work)
