# Supplementary material for: Health-care leaders’ experiences of the competencies required for crisis management during COVID-19: a systematic review of qualitative studies
Source: Leadersh Health Serv (Bradf Engl). 2023 May 11;36(4):595–610. doi: 10.1108/LHS-10-2022-0104 (PMC10853848; doi:10.1108/LHS-10-2022-0104)
Supplement: Supplementary file 2 [file leadershhealthserv-36-0595-s002.docx]

Supplementary Table 1 Data extraction

| Citation (First author, year, country) | Purpose | Participants | Methodology (Study setting, data collection, data analysis) | Key findings | Critical appraisal |
| --- | --- | --- | --- | --- | --- |
| Abu Mansour *et al.* (2022), Jordan | To explore the experiences of Jordanian first-line nurse managers during COVID-19. | Sixteen first-line nurse managers. | Four different types of hospital treating COVID-19 patients, including governmental hospitals, military, and private hospitals located in Zarqa and Amman cities. A qualitative descriptive phenomenological study. Semi structured face-to-face interviews. Phenomenological data analysis. | Four themes: Unprecedented pressure, Strengthening system and resilience, Building a supportive team, Maturity during the crisis. | 8/10 |
| Hølge-Hazelton *et al.* (2021), Denmark | To elaborate on the experiences of nursing ward managers during and after COVID-19, to reflect and learn how person-centred nursing leadership may be strengthened in future times of crisis. | Thirteen ward managers. | University Hospital in Denmark. The theoretical perspective was person-centred leadership. The study was a descriptive study using interviews via a semi structured interview guide. Telephone interviews three months after the first national COVID-19 case was confirmed. A qualitative directed content analysis. | Five themes and their subthemes: Leadership beliefs and values (Maintaining presence, own leadership virtues and professionalism; Designating and transferring staff; Practicing distance leadership), Sense of community (Sense of community at unit level; Sense of community at ward manager level; Sense of community at executive level), Balancing different stakeholder needs (Staff needs versus overall needs; Loyalty to own leaders versus staffs need; Upholding guidelines versus relatives’ and patients’ needs), Involvement in decision-making (Top-down decision-making; Bottom-up decision-making), Personal development (Being on a steep learning curve; Management education gives valuable tools; Personal development - Rewound). | 8/10 |
| Jackson *et al.* (2021), Canada | To understand the experiences of nurse managers during COVID-19. | Eight nurse managers. | Acute care and outpatient settings in Canada and USA. Medical-surgical areas, palliative care and outpatient services. Qualitative interview study, semi structured interviews that were conducted via Teams and Zoom. Only the audio of the interviews was recorded. Thematic analysis. | Four themes and their subthemes: The COVID context (Planning during uncertainty; Navigating the political climate), Changing the nurse manager role (Expanding roles and responsibilities; Changing roles and responsibilities), Managing transitions (Patients transitioning through the healthcare system; Workplace transitions in response to COVID-19; Maintaining quality through problem-solving), Nurse manager experiences of COVID-19. | 8/10 |
| Losty *et al.* (2021), USA | To explore current challenges of COVID-19 on health care delivery and leadership styles of nurse executives that were specific to the pandemic. To ascertain the essence of nurse executive leadership and innovation during COVID-19. | Six nurse executives. | USA. Descriptive qualitative study. Interviews were conducted via Web conference using preformatted guide. Thematic analysis. | Three themes: Communication is paramount; Having a leadership presence; Mental toughness. | 8/10 |
| Riddell *et al.* (2022), Australia | To understand the nursing response to COVID-19 in Melbourne, Australia from the perspective of nursing executives. Specifically, to explore the context in which nursing executives were working, nursing's contribution to the healthcare response and the impact from delivering healthcare services in response to the pandemic. | Fourteen executive-level nurse leaders. | General and specialist hospitals within metropolitan Melbourne. A qualitative, retrospective research design, with a constructivist approach. In-depth individual interviews were conducted via Zoom using a semi-structured interview guide. Reflexive thematic analysis conducted inductively, no a priori framework. | Four themes and their subthemes: rapid, relentless, around the clock action required (preparation insufficient; extensive information and communication flow; expanded working relationships; constant change; organizational barriers removed), nursing's multi-faceted contribution (leadership activities; flexible work approach and practices; knowledge development and dissemination; new models of care; workforce numbers), unintended consequences (negative experiences/experience of addressing the pandemic; mix of emotions; difficult personal and work conditions; negative outcomes for executives and workforce), silver linings (expanded ways of working; strengthened clinical practice; new opportunities; deepened working relationships). | 8/10 |
| Roche *et al.* (2021), UK | To explore the experiences of clinical leads in paediatric critical care units in England and Wales during the reorganisation of services in the initial surge of SARS-CoV-2 and to learn lessons for future surges and service planning. | Paediatric critical care consultant clinical leads and nurse leads. The number of participants was not disclosed as a specific number. | Paediatric critical care units in England and Wales during the reorganisation of services in the initial surge of SARS-CoV-2. A qualitative study design using semi structured interviews via virtual conferencing using an interview guide. Thematic analysis. | Six themes: leadership, management and planning; communication; workforce development and training; innovation; workforce experience; infection prevention and control. | 8/10 |
| Vázquez-Calatayud *et al.* (2022), Spain | To explore experiences of frontline nurse managers during COVID-19. | Ten frontline nurse managers. | Various units and services of a highly specialized university hospital in Spain. A qualitative descriptive study, semi-structured interviews. The audio of the interviews was recorded. Systematic analysis. | Six themes and their subthemes: constant adaptation to change (urgent and constant reorganization of the service; complexity of staff management in a changing situation; communication problems in changing situations), participation in decision-making, management of uncertainty, prioritization of the biopsychosocial well-being of the staff, preservation of humanized care, ‘one for all’ (teamwork; collaboration). | 8/10 |
| White (2021), USA | To understand the experiences of hospital nurse managers and assistant nurse managers during COVID-19 in the USA. | Thirteen participants, seven nurse managers and six assistant nurse managers. | Three hospitals in one large health care system in the Mid Atlantic area of USA. Critical care and medical surgical units that were converted to COVID-19 patient units. Interpretive phenomenology, semi structured interviews. The audio of the teleconference interviews was recorded. Interpretive phenomenological analysis. | Four themes and their subthemes: Being there for everyone (Carrying the burden; Reliance on me), Leadership challenges (A different kind of support; Revamping my approach; Staff's resistance and fears), Struggles, support and coping (Physical and emotional toll; Professional support; Personal coping), Strengthening my role (Reflections on learning; Rewarding influences; Work that needs attention going forward). | 8/10 |

(Source: Authors own work)
