# Supplementary material for: Health-care leaders’ experiences of the competencies required for crisis management during COVID-19: a systematic review of qualitative studies
Source: Leadersh Health Serv (Bradf Engl). 2023 May 11;36(4):595–610. doi: 10.1108/LHS-10-2022-0104 (PMC10853848; doi:10.1108/LHS-10-2022-0104)
Supplement: Supplementary file 3 [file leadershhealthserv-36-0595-s003.docx]

Supplementary Table 2 Search strategies in searched databases and their results

| Database | Search strategy | Results |
| --- | --- | --- |
| Scopus (Elsevier) | ( ( TITLE-ABS-KEY ( leader ) OR TITLE-ABS-KEY ( leaders ) OR TITLE-ABS-KEY ( manager* ) ) ) AND ( ( TITLE-ABS-KEY ( crisis ) OR TITLE-ABS-KEY ( crises ) OR TITLE-ABS-KEY ( disaster* ) OR TITLE-ABS-KEY ( emergenc* ) OR TITLE-ABS-KEY ( pandemi* ) ) ) AND ( ( TITLE-ABS-KEY ( competen* ) OR TITLE-ABS-KEY ( knowledge ) OR TITLE-ABS-KEY ( skill* ) OR TITLE-ABS-KEY ( attribute ) OR TITLE-ABS-KEY ( attitude* ) OR TITLE-ABS-KEY ( expertise ) OR TITLE-ABS-KEY ( knowhow ) OR TITLE-ABS-KEY ( capabilit* ) OR TITLE-ABS-KEY ( capacit* ) OR TITLE-ABS-KEY ( qualification* ) OR TITLE-ABS-KEY ( abilit* ) ) ) AND ( ( TITLE-ABS-KEY ( "health care" ) OR TITLE-ABS-KEY ( healthcare ) ) ) | 2,230 |
| PubMed (MEDLINE) | ("Nurse Administrators"[MeSH Terms] OR "leader"[Text Word] OR "leaders"[Text Word] OR "manager*"[Text Word]) AND ("Disease Outbreaks"[MeSH Terms] OR "Natural Disasters"[MeSH Terms] OR "crisis"[Text Word] OR "crises"[Text Word] OR "disaster*"[Text Word] OR "emergenc*"[Text Word] OR "pandemi*"[Text Word]) AND ("Professional Competence"[MeSH Terms] OR "Decision Making"[MeSH Terms] OR "competen*"[Text Word] OR "knowledge"[Text Word] OR "skill*"[Text Word] OR "attribute"[Text Word] OR "attitude*"[Text Word] OR "expertise"[Text Word] OR "knowhow"[Text Word] OR "capabilit*"[Text Word] OR "capacit*"[Text Word] OR "qualification*"[Text Word] OR "abilit*"[Text Word]) | 4,235 |
| CINAHL (EBSCO) | ( ( ( (MH "Leaders+") OR (MH "Nurse Administrators+") ) ) OR ( leader OR leaders OR manager* ) ) AND ( ( ( (MH "Disease Outbreaks+") OR (MH "Natural Disasters+")) ) OR ( crisis OR crises OR disaster* OR emergenc* OR pandemi* ) ) AND ( ( ( (MH "Professional Competence+") OR (MH "Decision Making+") ) ) OR ( competen* OR knowledge OR skill* OR attribute OR attitude* OR expertise OR knowhow OR capabilit* OR capacit* OR qualification* OR abilit* ) ) | 2,711 |
| ABI/INFORM (ProQuest) | noft(leader OR leaders OR manager*) AND noft(crisis OR crises OR disaster* OR emergenc* OR pandemi*) AND noft(competen* OR knowledge OR skill* OR attribute OR attitude* OR expertise OR knowhow OR capabilit* OR capacit* OR qualification* OR abilit*) AND noft("health care" OR healthcare) | 1,862 |
| Medic (Finnish Database) | kriisi* cris* AND johtami* leader* manage* | 9 |
| MedNar | (leader* OR manager*) AND (crisis OR crises OR disaster* OR emergenc* OR pandemi*) AND (competen* OR knowledge OR skill* OR attribute OR attitude* OR expertise OR knowhow OR capabilit* OR capacit* OR qualification* OR abilit*) AND (“health care” OR healthcare) | 846 |
| EBSCO Open Dissertations | (leader* OR manager*) AND (crisis OR crises OR disaster* OR emergenc* OR pandemi*) AND (competen* OR knowledge OR skill* OR attribute OR attitude* OR expertise OR knowhow OR capabilit* OR capacit* OR qualification* OR abilit*) AND (“health care” OR healthcare) | 62 |
| Searches conducted 13th March 2022, limited to English, Finnish or Swedish, no time limit.  (Source: Authors own work) | | |
