# Supplementary material for: Health-care leaders’ experiences of the competencies required for crisis management during COVID-19: a systematic review of qualitative studies
Source: Leadersh Health Serv (Bradf Engl). 2023 May 11;36(4):595–610. doi: 10.1108/LHS-10-2022-0104 (PMC10853848; doi:10.1108/LHS-10-2022-0104)
Supplement: Supplementary file 4 [file leadershhealthserv-36-0595-s004.docx]

Supplementary Table 3 List of excluded studies

| Study | Reason for exclusion |
| --- | --- |
| Abdi *et al.* (2022) Role of hospital leadership in combating the COVID-19 pandemic | Not a qualitative study: not a qualitative interview study. |
| Agee (2020) Nursing Leadership Amid a Pandemic | Not a qualitative study: commentary. |
| Albanese & Paturas (2018) The importance of critical thinking skills in disaster management | Not a qualitative study: not a qualitative interview study. |
| Aliakbari *et al.* (2021) Development, Psychometric Testing, and Use of a Disaster Nursing Competency Scale in Iran: A Mixed Methods Study | Includes additional participants who cannot be differentiated: nurses. |
| Bijani *et al.* (2021) Exploring senior managers' perceptions of the COVID-19 Crisis in Iran: a qualitative content analysis study | Includes additional participants who cannot be differentiated: included management positions outside of healthcare. |
| Cariaso-Sugay *et al.* (2021) Nurse Leaders' Knowledge and Confidence Managing Disasters in the Acute Care Setting | Not a qualitative study: quantitative study. |
| Cwiak *et al.* (2017) Emergency management leadership in 2030: Shaping the next generation meta-leader | Not a qualitative study: not a qualitative interview study. |
| Deitchman (2013) Enhancing crisis leadership in public health emergencies | Not a qualitative study: commentary. |
| Edmonson *et al.* (2016) The Nurse Leader Role in Crisis Management | Not a qualitative study: not a qualitative interview study. |
| Ernst (2020) Leading in crisis | Not a qualitative study: editorial. |
| Feldmann-Jensen *et al.* (2019) The next generation core competencies for emergency management | Not a qualitative study: not a qualitative interview study. |
| Guo (2003) A study of the skills and roles of senior-level health care managers | Not a qualitative study: does not include quotations from research participants and focuses on high-level leaders, not crisis management. |
| Harrington (2021) Understanding effective nurse leadership styles during the COVID-19 pandemic | Not a qualitative study: not a qualitative interview study. |
| Havaei *et al.* (2020) Leading a Long-Term Care Facility through the COVID-19 Crisis: Successes, Barriers and Lessons Learned | Not a qualitative study: mixed methods and focus on framework. |
| Hopkinson & Jennings (2021) Nurse Leader Expertise for Pandemic Management: Highlighting the Essentials | Not a qualitative study: case study & does not include quotations from research participants. |
| James & Bennett (2020) Effective nurse leadership in times of crisis | Not a qualitative study: framework. |
| Jónsdóttir *et al.* (2021) “There was no panic”—Nurse managers’ organising work for COVID-19 patients in an outpatient clinic: A qualitative study | Quotations of respondents were not indicated by a unique participant ID. |
| Kagan *et al.* (2021) A Mixed‐Methods Study of Nurse Managers' Managerial and Clinical Challenges in Mental Health Centers During the COVID‐19 Pandemic | Not a qualitative study: mixed methods; the qualitative side of the study did not focus on competencies. |
| Karabacak *et al.* (2011) Crisis Management: The Activities Of Nurse Managers in Turkey | Not a qualitative study: quantitative study. |
| Lefort *et al.* (2020) Crisis management in the face of SARS-CoV-2: management and leadership in health care | Full text not available in English, Swedish or Finnish. |
| Libsack (2021) Resourcefulness: A Key Leadership Skill: In a crisis, leaders need this competency even more | Not a qualitative study: column. |
| Macasteb (2021) Clinical Nurse Leaders' impact During the COVID-19 Pandemic | Not a qualitative study: opinion paper. |
| Morse & Warshawsky (2021) Nurse Leader Competencies: Today and Tomorrow | Not a qualitative study: not a qualitative interview study. |
| Nijjar (2020) Crisis Leadership: Lessons from the Front Line | Full text not available. |
| Paixão *et al.* (2020) Leadership in a crisis: doing things differently, doing different things | Full text not available. |
| Porche (2009) Emergent leadership during a natural disaster: A narrative analysis of an acute health care organization's leadership | Includes additional participants who cannot be differentiated: includes participants other than line managers. |
| Reyes *et al.* (2021) Translating 6 key insights from research on leadership and management in times of crisis | Not a qualitative study: not a qualitative interview study. |
| Shabanikiya *et al.* (2016) Assessment of Hospital Management and Surge Capacity in Disasters | Does not include quotations from research participants. |
| Shih *et al.* (2009) Surviving a life-threatening crisis: Taiwan's nurse leaders' reflections and difficulties fighting the SARS epidemic | Quotations of respondents were not indicated by a unique participant ID. |
| Shuman & Costa (2020) Stepping in, stepping up, and stepping out: competencies for intensive care unit nursing leaders during disasters, emergencies, and outbreaks | Not a qualitative study: commentary. |
| Smith & Bhavsar (2021) A new era of health leadership | Not a qualitative study: not a qualitative interview study. |
| Smithson (2021) The compatibility of multiple leadership styles in responding to a complex crisis: leading a health service COVID-19 response | Does not include quotations from research participants. |
| Standiford *et al.* (2021) Physician leadership during the COVID-19 pandemic: An emphasis on the team, well-being and leadership reasoning | Includes additional participants who cannot be differentiated: emerging leaders. |
| Tseng *et al.* (2005) SARS: key factors in crisis management | Study purpose not suitable: the focus is on a hospital level and not on the individuals’ competencies. |
| Veenema (2017) Hospital administration and nursing leadership in disasters: An exploratory study using concept mapping | Does not include quotations from research participants. |

(Source: Authors own work)
