# Supplementary material for: Health-care leaders’ experiences of the competencies required for crisis management during COVID-19: a systematic review of qualitative studies
Source: Leadersh Health Serv (Bradf Engl). 2023 May 11;36(4):595–610. doi: 10.1108/LHS-10-2022-0104 (PMC10853848; doi:10.1108/LHS-10-2022-0104)
Supplement: Supplementary file 5 [file leadershhealthserv-36-0595-s005.docx]

Supplementary Table 4 Critical appraisal results for included studies using the JBI-Qualitative Critical Appraisal Checklist (Lockwood et al., 2015)

| Study | Q1 | Q2 | Q3 | Q4 | Q5 | Q6 | Q7 | Q8 | Q9 | Q10 |
| --- | --- | --- | --- | --- | --- | --- | --- | --- | --- | --- |
| Abu Mansour & Abu Shosha (2022) | Y | Y | Y | Y | Y | U | U | Y | Y | Y |
| Hølge-Hazelton *et al.* (2021) | Y | Y | Y | Y | Y | U | U | Y | Y | Y |
| Jackson & Nowell (2021) | Y | Y | Y | Y | Y | U | U | Y | Y | Y |
| Losty & Bailey (2021) | Y | Y | Y | Y | Y | U | U | Y | Y | Y |
| Riddell *et al.* (2022) | Y | Y | Y | Y | Y | U | U | Y | Y | Y |
| Roche *et al.* (2021) | Y | Y | Y | Y | Y | U | U | Y | Y | Y |
| Vázquez-Calatayud *et al.* (2022) | Y | Y | Y | Y | Y | U | U | Y | Y | Y |
| White (2021) | Y | Y | Y | Y | Y | U | U | Y | Y | Y |

Y - Yes, N - No, U - Unclear, N/A - not applicable

Q1 Is there congruity between the stated philosophical perspective and the research methodology?

Q2 Is there congruity between the research methodology and the research question or objectives?

Q3 Is there congruity between the research methodology and the methods used to collect data?

Q4 Is there congruity between the research methodology and the representation and analysis of data?

Q5 Is there congruity between the research methodology and the interpretation of results?

Q6 Is there a statement locating the researcher culturally or theoretically?

Q7 Is the influence of the researcher on the research, and vice- versa, addressed?

Q8 Are participants, and their voices, adequately represented?

Q9 Is the research ethical according to current criteria or, for recent studies, and is there evidence of ethical approval by an appropriate body?

Q10 Do the conclusions drawn in the research report flow from the analysis, or interpretation, of the data?

(Source: Authors own work)
