# Supplementary material for: Health-care leaders’ experiences of the competencies required for crisis management during COVID-19: a systematic review of qualitative studies
Source: Leadersh Health Serv (Bradf Engl). 2023 May 11;36(4):595–610. doi: 10.1108/LHS-10-2022-0104 (PMC10853848; doi:10.1108/LHS-10-2022-0104)
Supplement: Supplementary file 6 [file leadershhealthserv-36-0595-s006.docx]

Supplementary Table 5 Correspondence between study findings and illustrations provided

| Study | Findings | Illustrations | Level of credibility |
| --- | --- | --- | --- |
| Abu Mansour & Abu Shosha (2022) | Colleagues’ support | We all support each other and work as a team. Everyone was working … the nursing administration, quality unit team, infection control team, and general safety team. We all worked to control the pandemic. (Participant 1) (Abu Mansour & Abu Shosha, 2022) p.389 | C |
|  | Compensating for shortage of materials and human resources | Modifying the emergency plan that by studying the entire situation and reviewing the neighboring countries’ emergency plans … we could deal better with the pandemic. (Participant 9) (Abu Mansour & Abu Shosha, 2022) p.388 | C |
|  | Extraordinarily demanding system | Our working hours and the pressure increased as we are the first-line of defense against this virus. We have to be available 24 hours during the week. The nature of the shifts has changed …. For example, the eight-hour shift changed to a 12-hours shift. (Participant 9) (Abu Mansour & Abu Shosha, 2022) pp.386-387 | C |
|  |  | Besides the shortage of nurses, we were not sufficiently trained to deal with such diseases. So, we had faced many problems but overtime nurses gained the appropriate experience. (Participant 8) (Abu Mansour & Abu Shosha, 2022) p.387 | C |
|  | Maturity of management skills | As a nurse manager and member of the crisis team formed inside the hospital, the hospital administrators gave us the green light to make decisions… For example, the crisis team suggested using the elevators only by staff working in isolation units … we immediately got the approval from the hospital administrator. (Participant 1) (Abu Mansour & Abu Shosha, 2022) p.389 | C |
|  | Physically demanding situations | Physically, it was exhausting. I had fatigue because I went home late a lot. I worked for 12 to 15 hours. I was deprived of vacations. (Participant 6) (Abu Mansour & Abu Shosha, 2022) p.387 | U |
|  | Self-awareness | Despite the difficult and demanding situation of COVID-19, I have a strong commitment to work …. I did my job excellently …. I felt responsible and accountable as a nurse manager. (Participant 8) (Abu Mansour & Abu Shosha, 2022) p.389 | U |
|  | Staff development | We have been trained with the new equipment and are constantly provided with new issues related to the disease after receiving sufficient information and comprehensive instructions. As nurse managers, we communicated with the staff to convey information and enhance their awareness of the disease. (Participant 3) (Abu Mansour & Abu Shosha, 2022) p.388 | C |
|  |  | Due to the imposition of social distancing rules, the lecture halls of the hospital were closed, and hence, it was difficult to conduct in-person workshops. Thus, workshops and seminars were conducted virtually. (Participant 7) (Abu Mansour & Abu Shosha, 2022) p.388 | C |
| Hølge-Hazelton *et al.* (2021) | Being on a steep learning curve | I have learned a lot and I would not do it the same way next time. I would have more dialogue with the staff. I have been much less empathetic and much tougher than I usually am, and I will strive to change that balance. (WM-C) (Hølge-Hazelton *et al.*, 2021) p.1408 | U |
|  | Bottom-up decision-making | When I stood with my back against the wall, I decided to establish a steering group with the hygiene nurse, an IT- super-user and our quality assurance nurse. I said: now it is you, it is us, who take decisions, all staff can give their inputs, but it is this group that decides, and the staff thought it was a super solution. (WM-H) (Hølge-Hazelton *et al.*, 2021) p.1407 | U |
|  |  | We quickly agreed to meet every Friday: how is this done nationally? (..) We gave each other practical advice in a situation where everything came top down and you felt you ran your forehead into the wall. When I realized what they did in other places, and had this kind of sparring I was able to lead a little upwards. (WM-L) (Hølge-Hazelton *et al.*, 2021) p.1408 | C |
|  | Designating and transferring staff | My own values have been overruled. The staff were frightened, nervous and crying, but I had to use my professional side not my emotional side when deciding whom to transfer to COVID-19 units (..) and I have been much less empathetic - and much more tough. I would like to strive to change this balance ... but I did not have room or time for discussions in the acute situation. (WM-C) (Hølge-Hazelton *et al.*, 2021) p.1405 | U |
|  | Loyalty to own leaders versus staff need | I have missed that my nearest leaders contacted and informed me about what went on. Loyalty has sometimes been difficult because you do not feel that you get the knowledge you need. (WM-A) (Hølge-Hazelton *et al.*, 2021) p.1407 | C |
|  | Maintaining presence, own leadership virtues and professionalism | I had the phone under the pillow at night as the employees should to be able to call at all hours of the day - and they did. (WM-G) (Hølge-Hazelton *et al.*, 2021) p.1405 | U |
|  |  | It is one of my values to be open about I do not know it all. I am very humble and aware of staff who know more than I and I often say: I honestly don’t know the answer to this, but let us investigate it or ask a friend. (WM-D) (Hølge-Hazelton *et al.*, 2021) p.1405 | U |
|  | Management education gives valuable tools | My Master-education has been worth gold; you meet others and understand frustrations better. It has given me tools to understand this situation. I have been better at explaining to the staff. You learn to reflect at a different level, to keep calm, and use your network. (..) Through education you learn to use networks, and that it is ok not to know the answer to everything, you need to draw on others. (WM-L) (Hølge-Hazelton *et al.*, 2021) p.1408 | U |
|  | Personal-development - rewound | I do not know if it has developed me as a leader. I feel like it has degraded me in many ways, as I could not be the leader I wished to be. I suddenly became an advanced shift planner. (WM-G) (Hølge-Hazelton *et al.*, 2021) p.1408 | C |
|  |  | On a personal level this (pandemic) has given much, I have never tried something like this before. It has given me experiences on where I do not wish to be in leadership and what I will tolerate (..) Perhaps I need to learn to say no, but it is difficult as a new leader to say no and that enough is enough. (WM-E) (Hølge-Hazelton *et al.*, 2021) p.1408 | C |
|  |  | It could have been helped with education and experience; I just started my leadership education and had to stop it. I have no experience and I have acted solely on my gut feeling. (WM-E) (Hølge-Hazelton *et al.*, 2021) p.1408 | C |
|  | Practicing distance leadership | My role has been very unclear. How much leadership was I responsible for? Should I lead the transferred nurses who were sad and contacted me? (WM-D) (Hølge-Hazelton *et al.*, 2021) p.1405 | C |
|  |  | The leadership I usually practice has been set on hold. It is difficult to be leader for someone you don’t know, and you are alone on the post and you get a new group of staff every fortnight. Suddenly my working week lasted 70 hours because I prioritized taking care of the staff. (WM-G) (Hølge-Hazelton *et al.*, 2021) p.1405 | C |
|  | Staff needs versus overall needs | My staff had to be transferred and they felt insecure, overwhelmed and insufficient. (WM-I) (Hølge-Hazelton *et al.*, 2021) p.1406 | C |
|  |  | I had to defend and transfer the information to my staff and it made no sense. So I just did it, I stopped reflecting on how and why - the orders were as they were. (WM-F) (Hølge-Hazelton *et al.*, 2021) p.1407 | C |
|  |  | The insecurity about how to best protect ourselves has taken up a lot of time. The patients should be treated well, but we also needed to protect ourselves. (WM-A) (Hølge-Hazelton *et al.*, 2021) p.1407 | C |
|  | Upholding guidelines versus relatives and patients’ needs | One of the biggest leadership challenges has been to take the relatives needs into account. The limited access of relatives has led to conflicts that I have taken responsibility for. (WM-M) (Hølge-Hazelton *et al.*, 2021) p.1407 | C |
|  |  | The restrictions with relatives’ present have led to some conflicts with relatives which I have undertaken. One bad-mouthed me and called me various things but I’m pretty cool with that, there was not much else to do. (WM-M) (Hølge-Hazelton *et al.*, 2021) p.1407 | C |
| Jackson & Nowell (2021) | Changing roles and responsibilities | So, I was doing probably 4 different jobs. I lost my admin because she went to [facility]. I lost my intake nurse in hospice. And I lost a couple of my nurses, and my social worker was working from home, and we had no other people allowed in, or any of our other therapists. So, yeah, I mean I was doing everything from cooking lunch, because we have a kitchen that usually the volunteers use to make food for patients, to being a bedside nurse, to doing all the referrals and administrative stuff (M6). (Jackson & Nowell, 2021) p.2396 | C |
|  | Maintaining quality through problem-solving | ‘Everybody over the age of two has to wear a mask [...] Those large surgical masks do not fit a kid. So, we created a video on how to tie the knot [to shorten the mast straps] (M4) (Jackson & Nowell, 2021) p.2397 | C |
|  | Navigating the political climate | Everyone was still going into the office that I reported to. And it was very much. ‘Oh, this is a hoax. We do not know why people are getting crazy. This is all a hoax. The President said it will be fine. People just need to stop panicking. (M3) (Jackson & Nowell, 2021) p.2395 | C |
|  | Patients transitions through the healthcare system | How do we get them out of this unit safely to the front doors, anticipating that their loved one who’s going to pick them up, they completely understand about the quarantine self isolation, the cleaning that’s required, the hand hygiene, picking up their medications for discharge in anticipation of them going home (M7). (Jackson & Nowell, 2021) p.2396 | C |
|  | Planning during uncertainty | I do not think we all thought that it was going to get to where everybody was being self isolated and quarantined. And then no visitors in the hospital, and stuff like that. I do not think we ever thought we’d get to that level, not here (M8). (Jackson & Nowell, 2021) p.2395 | C |
|  |  | So, there was absolutely nothing. No documentation, no training, they [people responding to staff cases] were all just nurses from all over the organizations and no one had an occupational health background. No one knew how to deal with an outbreak. What are you supposed to do? (M2). (Jackson & Nowell, 2021) p.2395 | C |
|  |  | When they made a decision on Tuesday night that every employee will wear PPE beginning tomorrow morning at eight o’clock. Some buildings did not have enough PPE for the people in them. So, that was frightening for the nurses, it was frightening for any healthcare provider. Do I get a mask? [as a nurse manager] I do not get a mask today, then they gave me a mask and by four o’clock he told me to take my mask off and I’ll get a mask next week. What’s different, why am I different? Why am I being denied my PPE? (M7) (Jackson & Nowell, 2021) p.2395 | C |
|  |  | ‘It’s definitely introduced new ideas about how work can be done. And I think some of those ideas, like virtual care is one of them (M1) (Jackson & Nowell, 2021) p.2395 | C |
|  | Workplace transitions in response to COVID-19 | ‘We had to reschedule 4000 or more patients (M5) (Jackson & Nowell, 2021) p.2396 | C |
|  |  | At one point before COVID, we were not allowed to buy paper pretty much anymore because our budget was so tight. And then you see this money going to the stuff that might not happen [pandemic contingency plans] and you know as a manager that the budget fallout of that is going to be huge (M5). (Jackson & Nowell, 2021) pp.2396-2397 | C |
| Losty & Bailey (2021) | Communication is paramount | We needed to understand what COVID-19 was and how to respond to the quickly evolving situation. We developed 209 policies and procedures in less than 45 days. Our success rested upon effective and frequent communication. (NE01) (Losty & Bailey, 2021) p.120 | C |
|  |  | Our city was one of the hotspots in the country for COVID-19 and between 60% and 70% of our patients required an ICU level of care. This situation created an “all hands-on deck” situation that required good communication. (NE03) (Losty & Bailey, 2021) p.120 | C |
|  |  | We established a hotline for our community to call for questions and we established curbside testing centers at each campus. We communicated on all fronts and rose to the occasion. (NE03) (Losty & Bailey, 2021) p.120 | C |
|  |  | Our first challenge involved managing the frightening images portrayed by the media that did not initially materialize in our area. To address this, senior leaders at the system and local levels used various technologies to maximize communication with staff and patients. (NE04) (Losty & Bailey, 2021) p.120 | C |
|  |  | Our organization is steeped in relationship-based care and with COVID-19, we went from being a family-centered care model to a no-visitor organization. By working closely with the information technology team, nurses established a “no touch Zoom” option for not only families but for staff and physicians, which allowed for multiple individuals to connect with the patient. (NE01) (Losty & Bailey, 2021) pp.120-121 | C |
|  | Having a leadership presence | Our priorities focused on addressing the challenges of our staff while they were providing care to our patients and their families. It was like building a plane and flying it at the same time. (NE01) (Losty & Bailey, 2021) p.121 | C |
|  |  | Personally, I did have some apprehension that I would not be able to meet the staff’s needs or give them the tools they needed to be successful. I learned to let the staff that I work with lead as well. (NE02) (Losty & Bailey, 2021) p.121 | C |
|  |  | I quickly recognized that my job was to get out of the way as COVID-19 provided staff the opportunity to create teams, to collaborate with members from other disciplines, and to create new roles to respond to what they needed. (NE02) (Losty & Bailey, 2021) p.121 | C |
|  |  | Part of being a leader was allowing the nurses to make decisions that affected THEIR WORK. I learned that I needed to LISTEN to my frontline and provide them with the support and trust during these difficult times. (NE03) (Losty & Bailey, 2021) p.121 | C |
|  |  | Our success in dealing with COVID-19 resulted from the flexibility of the nursing leadership in being leaders and being followers. (NE05) (Losty & Bailey, 2021) p.121 | C |
|  |  | Our team embraced the crisis as there is an expectation for us to be successful! The leaders’ challenge was to create ways to maintain this spirit among our staff (NE06). (Losty & Bailey, 2021) p.121 | C |
|  | Mental toughness | As the supply chain became challenged, one nurse developed a face shield that connected to her glasses using a magnet; it was amazing how innovative and flexible the nurses became when faced with uncertainty. (NE01) (Losty & Bailey, 2021) p.121 | C |
|  |  | Our nurses embraced the situation and rose to the occasion. For example, one of our RNs who was being interviewed by a major news network questioned herself, as a professional, if she were working enough and wanted to know what else she could do to give back. I was humbled by her professional commitment. (NE02) (Losty & Bailey, 2021) pp.121-122 | C |
|  |  | The nurses maintained their dedication to the organization’s mission and supported their community and their patients. As leaders, we worried about the psychological crisis that may ensue and how best to encourage the nurses to take a break. We found that they did not want to take a break. (NE02) (Losty & Bailey, 2021) p.122 | C |
| Riddell *et al.* (2022) | Expanded ways of working | We can stand up a pop-up screening clinic in about four hours now. We've got it down so pat. It's amazing what we can do. That kind of agility is pretty impressive, and we don't want to lose it. (Participant 006) (Riddell *et al.*, 2022) p.12 | C |
|  |  | “hitting the ground running” (Participant 002) (Riddell *et al.*, 2022) p.12 | C |
|  | Expanded working relationships | We still meet as an executive team daily. One of the things that we received a lot of really positive feedback for was that people said they really appreciated the rapid decision-making that meeting daily provided and people didn't feel they were having to wait. (Participant 014) (Riddell *et al.*, 2022) pp.6-7 | C |
|  | Extensive information and communication | ...so there were multiple sources of information, some of them more reliable than others, that was just leading to great confusion to be quite honest. (Participant 008) (Riddell *et al.*, 2022) p.6 | C |
|  | Flexible work approach and practices | We were probably four to six weeks ahead of the Department. So, we would implement things and then four to six weeks later they would put out a policy saying ‘This is how it's going to be’ ... but we wouldn't sit on our hands, I suppose, and wait for that to come through. (Participant 008) (Riddell *et al.*, 2022) p.8 | C |
|  |  | If we'd waited for the guidelines, we wouldn't have had those positions [COVID safety spotters] to keep our staff safe. (Participant 013) (Riddell *et al.*, 2022) p.8 | C |
|  |  | Everything that came out was almost too late. (Participant 005) (Riddell *et al.*, 2022) p.8 | C |
|  |  | ...to the point that - and you'll no doubt hear this from others - I was in scrubs. I was giving care. I was washing and feeding. It's been a while since I've been on the tools. (Participant 002) (Riddell *et al.*, 2022) p.9 | C |
|  |  | ...and pulling massive hours, to get it all - stay on top of it all. Plus running a hospital, as well. It's not just all about COVID [laughs]. We still had to run the health services. (Participant 009) (Riddell *et al.*, 2022) p.9 | C |
|  |  | I have a strong belief in visible leadership, okay. All of a sudden, that visibility, how you led changed, because I couldn't be out there on the floor. I wasn't allowed to go on the red zones anymore and you know, I think that was challenging too. (Participant 001) (Riddell *et al.*, 2022) p.9 | C |
|  | Knowledge development and dissemination | I think one of the things I'm really incredibly proud of is the training team that [we] stood up virtually overnight. That went out across the state, so that people could have some training in how to use PPE because it was a gap within the private aged care that we just had previously been blinded to. Where they had equipment, they didn't know how to use it, and oftentimes they didn't have equipment. (Participant 014) (Riddell *et al.*, 2022) p.9 | C |
|  |  | I was talking to the Chief Medical Officer for Australia and he was listening to me and being guided by me ... you know, I'm a [nursing executive-level role] working in a health service, and, no, I'm not normally in that position. (Participant 004) (Riddell *et al.*, 2022) p.9 | C |
|  | Removal of organizational barriers | Normally we're very consultative and to get policy changes takes probably about three months for them to go through all the various committees. It was just approved through incident command and put up that day. (Participant 006) (Riddell *et al.*, 2022) p.7 | C |
|  |  | We had thrown around a centralized recruitment model at some point [prior to COVID-19]. We had kind of a business case so we knew how it would work and we just stood it up, basically. (Participant 012) (Riddell *et al.*, 2022) p.7 | C |
| Roche *et al.* (2021) | Communication | “There were daily management meetings where information dissemination from the top and clinicians relaying frontline experience.” (pt 6) (Roche *et al.*, 2021) p.4 | C |
|  |  | “At start of the pandemic, we ensured that there was a sister in charge every shift (not the case before then). Lead nurse present every day.” (pt 11) (Roche *et al.*, 2021) p.4 | C |
|  | Innovation | “Psychology team very present, working remotely but made themselves available to everyone the same day.” (pt 12) (Roche *et al.*, 2021) p.4 | C |
|  |  | “We had to learn a whole new way of communicating with virtual wards rounds.” (pt 5) (Roche *et al.*, 2021) p.4 | C |
|  |  | “Brilliant changes with all the online stuff, webcam stuff, future world opportunities- less travel, environment, time benefits, safety.” (pt 4) (Roche *et al.*, 2021) p.4 | C |
|  |  | “Staff support for each other is biggest thing.” (pt 7) (Roche *et al.*, 2021) p.4 | C |
|  |  | “Interventions that were helpful for wellbeing were pre-shift chats for a couple of minutes - team spirit, everyone quite buoyed up.” (pt 17) (Roche *et al.*, 2021) p.4 | C |
|  | Leadership, management and planning | “Better for front line to be making decisions. With additional partitions ... easier for those who work there to explain what they were doing to others.” (pt 11) (Roche *et al.*, 2021) p.4 | C |
|  |  | “People from finance mopping the floors. People just got on with it.” (pt 2) (Roche *et al.*, 2021) p.4 | C |
|  |  | “The new way of working also worked well because it was an open system - people were able to speak up and say if something is not working. People learnt that they could speak up when needed.” (pt 17) (Roche *et al.*, 2021) p.4 | C |
|  |  | “There was detailed planning internally prior to pandemic, perhaps too much rigid planning. This ended up with changing plans on the fly as circumstance changed. May have benefitted from a flexible plan with clear authorisation plans (eg, who signs off) and some autonomy on the clinical level.” (pt 6) (Roche *et al.*, 2021) p.4 | C |
|  |  | “Empowered paediatric intensive care nurses to be tutors and mentors, really capable people who were acknowledged and generated a whole raft of educators who weren’t there before and gave them confidence. Helped junior [nurses] to develop leadership skills overseeing theatre staff - really shone.” (pt 1) (Roche *et al.*, 2021) p.4 | C |
|  | Workforce development and training | “As part of training system for non-critical care nursing staff, they would buddy up with Paediatric Intensive Care nurse and this worked well for new staff coming in.” (pt 1) (Roche *et al.*, 2021) p.4 | C |
|  |  | “Mock bed space made for simulation. Used for common scenarios. Anaesthetists didn’t require specific training, just local level induction.” (pt 2) (Roche *et al.*, 2021) p.4 | C |
|  |  | “What works to make everyone follow the guidance is reminders - nurses always very good at that. Two to three times daily donning/doffing training for staff. Posters outside with pictures of staff wearing standard PPE and face masks.” (pt 10) (Roche *et al.*, 2021) p.4 | C |
|  |  | “Teaching on ward rounds has almost disappeared due to the constraints of PPE.” (pt 3) (Roche *et al.*, 2021) p.4 | C |
|  |  | “With the advent of Zoom and MS Teams access to educational teaching is much better.” (pt 15) (Roche *et al.*, 2021) p.4 | C |
|  |  | “Variable transferability of skills ... a challenge was working out what people can and can’t do.” (pt 1) (Roche *et al.*, 2021) p.4 | C |
|  |  | “Nurses generally felt unsupported in unfamiliar working environment. Were expected to manage adult patients without induction/support.” (pt 8) (Roche *et al.*, 2021) p.4 | C |
| Vázquez-Calatayud *et al.* (2022) | Collaboration | We work at an institution where when you call, the doors open (..) but [it] also supports you with accompaniment (..). The support is impressive. (NM9) (Vázquez-Calatayud *et al.*, 2022) p.86 | C |
|  |  | And then, I can tell you that other services have had (..) a very good attitude (..). They have made everything easier. (NM10) (Vázquez-Calatayud *et al.*, 2022) p.86 | C |
|  |  | When I had needs or doubts ... I have always had a team that answered me at the moment and came and explained things to us. (NM2) (Vázquez-Calatayud *et al.*, 2022) p.86 | C |
|  |  | There are many things for which, at certain times, the response is, ‘Send me an email; write it down’. Well, no; now it is enough to call to get an answer, which I think has made things easier. In general, we have been open to not requiring written notification of authorisation to do something .... We have also been able to skip the usual regulatory channels to respond. (NM7) (Vázquez-Calatayud *et al.*, 2022) p.86 | C |
|  | Complexity of staff management in a changing situation | What has happened to us is that the staff changed every so often, between some being infected and then having to replace them and then the number of patients increased, because we had to bring new people, so it was difficult to introduce new people (..). That has added more work, perhaps, for management because you did not have much time to teach a lot to another person .... (NM10) (Vázquez-Calatayud *et al.*, 2022) p.83 | C |
|  |  | We were going to have to care for critical patients when the staff here (..) are not used to attending intubated patients; they do not know how to handle them, so they have helped us (..), people from the surgical area who were trained with ventilators (..), but that is an added fear (..) because patient care is the most important thing we have to provide. (NM3) (Vázquez-Calatayud *et al.*, 2022) p.83 | C |
|  | Managing uncertainty | You have to show you feel secure, even though you have doubts inside you, right? And this is so, but no matter how many times you try not to show your insecurity, in the end, you convey it. (NM9) (Vázquez-Calatayud *et al.*, 2022) p.84 | U |
|  |  | Convey calm; they have been telling me that everyone had the sense of everything being organised, like there was calm inside the chaos or uncertainty and fear. (NM6) (Vázquez-Calatayud *et al.*, 2022) p.84 | C |
|  |  | Each day you came to work, it was something different (..). Therefore, for us, the most important thing was to get ahead of events, that is, that they never caught us unaware. (NM4) (Vázquez-Calatayud *et al.*, 2022) p.84 | C |
|  | Participation in decision-making | ...our contributions have helped them, and they have listened to us (..); if we felt that something was not right, then we would go over it [occupational risks] again with them to change it ... they did not make the protocols [for occupational risks] alone; they made them with us.... (NM2) (Vázquez-Calatayud *et al.*, 2022) p.84 | U |
|  |  | Now, afterwards, I realise how many things we did in a few days and that we organised without considering anyone’s decisions (..). Therefore, you can see that it has been a time of making important and quick decisions without many people agreeing on them (..) and saying, ‘My goodness, this is me; here I am deciding this, and I would have liked to have been able to agree with three or four others, right?’ (NM1) (Vázquez-Calatayud *et al.*, 2022) p.84 | C |
|  |  | Some decisions are made without considering ..., without thinking about the repercussions they may have. I don’t know, it’s as if ... if everyone can speak, everyone is involved, I think it would be better. (NM5) (Vázquez-Calatayud *et al.*, 2022) p.84 | C |
|  |  | You did not know what to rely on to reinforce these regulations. Everything was changing so fast that, in the end, you saw that you were not capable of making a correct decision because you did not really know what the real criterion was for making that decision (..). So, I lacked some autonomy because, in the end, you do many things according to your decision, but you still want to have slightly more general information .... (NM7) (Vázquez-Calatayud *et al.*, 2022) p.84 | C |
|  | Preservation of humanized care | I did not know the patients. I did not recognise their faces. I only knew them by name ... Going through the unit and not being able to ... and not having their families ... that was really hard for me. (NM6) (Vázquez-Calatayud *et al.*, 2022) p.85 | C |
|  |  | Do not forget about the person. It has been very hard, the truth, because we have not been able to care as perhaps we would have liked to care, but that has been the greatest challenge: being able to meet all the needs of the patient with care that is a little different from usual. (NM10) (Vázquez-Calatayud *et al.*, 2022) p.85 | C |
|  |  | I have been able to accompany people who were dying to their last breath, who were alone, and I felt good because I was able to do that for them (..). There was also the challenge of keeping their families informed so that they would not be lost, not knowing where the relatives were or how they were admitted. (NM2) (Vázquez-Calatayud *et al.*, 2022) p.85 | C |
|  |  | We asked them if they had been able to speak with their family; we would facilitate a call or make it ourselves and give the patient the phone. (NM10) (Vázquez-Calatayud *et al.*, 2022) p.85 | C |
|  | Prioritization of the biopsychosocial well-being of staff | That the staff were comfortable working within the circumstances in which they lived (..), they had shifts so that they were well-rested when they came to work (..), that they had material, that they did not lack anything. (NM2) (Vázquez-Calatayud *et al.*, 2022) p.85 | C |
|  |  | Another important challenge was organising the groups of professionals. I made two groups in all the units so that they did not mix with each other and so that if there was a concern regarding infection in one, the other stayed, even though we worked with isolation protocols. (NM1) (Vázquez-Calatayud *et al.*, 2022) p.85 | C |
|  |  | When the shift groups were made, groups were not made randomly but with certain characteristics. Those groups were always the same. In addition, it was done with a thought towards like-minded people (..), and I think that was successful .... (NM6) (Vázquez-Calatayud *et al.*, 2022) p.85 | C |
|  |  | Therefore, making those groups and then also bringing in the people who came, if they were an experienced person in X [service], I put them in a group that lacked that (..). That’s how we went about figuring out groups. (NM3) (Vázquez-Calatayud *et al.*, 2022) p.85 | C |
|  |  | My priority with the staff was to make sure they didn’t lack anything; to listen to them in case someone didn’t feel well enough to work; to talk to them to give them more days off if they needed it, or to replace them with other people, so that they would be well, calm, not overwhelmed; (..) we used to get together to share the good things, the bad things, their fears. They were calmer when they talked and said what they thought. (NM2) (Vázquez-Calatayud *et al.*, 2022) p.85 | C |
|  |  | Changing people shifts, extending working hours (..), they said, ‘It’s that I have my parents, my children, but I work elsewhere’; what do we do? Of course, we are going to help you. We are going to do what we can together; we will arrange shifts. (NM6) (Vázquez-Calatayud *et al.*, 2022) p.85 | C |
|  | Teamwork | These moments have really united the team. They have worked phenomenally as a team; they have laughed, they cried together .... (NM2) (Vázquez-Calatayud *et al.*, 2022) p.86 | C |
|  |  | It has been very easy to manage all the changes that have been generated in staff members, shifts, rotations; that is, they have helped a lot; they have made it much easier to be able to make the protocols and change them continuously. (NM10) (Vázquez-Calatayud *et al.*, 2022) p.86 | U |
|  |  | The support (..), the team of three was very helpful because you feel supported, the decisions, the consultations ... I think it is a priority that as a team, we were going to direct, to organise as one, in synch, together, to get by, because there were many difficult moments. (NM6) (Vázquez-Calatayud *et al.*, 2022) p.86 | U |
|  |  | We have worked a lot as a team with the COVID team, which included infectious disease physicians, pneumologists ... we met every day and talked patient by patient about how we saw it and how we could improve. (NM2) (Vázquez-Calatayud *et al.*, 2022) p.86 | U |
|  | Urgent and constant reorganization of the service | When you are in the situation, you are so involved (..) in work, in organising (..). They called: ‘Hey, you have to organise this’, and there you are; it cannot wait for the next day, right? Things changed from one day to the next, even within hours, the protocols changed, and the procedures; so it was, well, a constant, constant, constant change (..). (NM1) (Vázquez-Calatayud *et al.*, 2022) p.83 | C |
|  |  | (..) what were we going to do? If not, we were looking for alternatives to things: well, if there were no such thing ... we would look for alternatives to many resources that were not available (..). We had to search for alternatives to the problems that came up and solve them quickly ... because, well, you are never prepared; you always have things, you always have resources, alternatives. (NM6) (Vázquez-Calatayud *et al.*, 2022) p.83 | C |
|  |  | Before the worst of the pandemic arrived, we already began to work on things; we tried to organise (..) with all the experience we had of what was happening, of what we were hearing from Madrid (..); we got ahead of many things (..), most importantly, especially staff, patient organisation, organisation of medical teams, equipment, training .... (NM6) (Vázquez-Calatayud *et al.*, 2022) p.83 | C |
| White (2021) | A different kind of support | My biggest concern and was just the emotional stuff the staff was going through. They would do post mortem care on one patient which was so emotional for them, and then immediately have to go to take care of all of the others they were assigned to. I would try to help them---make sure they took a break even for 5 minutes, or I would do their meds, anything I could to help them with their emotions. (ANM) (White, 2021) p.1530 | C |
|  | Personal coping | Coping was an issue. Usually I would go to the gym or exercise. I needed to feel better about myself. Working 12 or 16 hours a day did not leave me much time for that. I relied a lot on my wife. She was a god send. She was so patient. (NM) (White, 2021) p.1531 | U |
|  |  | While there were some interventions they set up for stress, these were not specific and took time that I did not have. I did use the Lavender Team a couple of times. I had to make sure I had time off to attend any programs and that was so difficult for all of us. (NM) (White, 2021) p.1531 | C |
|  | Professional support | I am so thankful for my staff. We work as a team. I appreciated even the little things that helped, like a staff member coming to clean a room. The team had to pitch in when ‘lines’ (IVs) needed to be started and we did not have enough providers who do this. (ANM) (White, 2021) p.1531 | C |
|  | Reflections on learning | One thing I realized that I will do differently was because in this role we are kind of autonomous. The staff does not usually see the big picture—the whole hospital. Now I see they need to be included in why decisions are made ---going forward I plan to do more of this. (NM) (White, 2021) p.1531 | U |
|  | Reliance on me | My job was 24/7. I went to work because I know the staff needed me. If I wasn’t there, I worried that they would not have what they needed and know about the new directives. At home, I was always texting them to see if things were okay. (NM) (White, 2021) p.1530 | U |
|  | Revamping my approach | Communication became a central focus of my work. It was constant I had to keep everyone informed— communication came from the top-down and was critical. Protocols were always in flux. We used texts, e-mails and written flyers to help. (NM) (White, 2021) p.1530 | C |
|  | Work that needs attention going forward | I don’t think nurses do such a good job of taking care of themselves. I’m making sure, now that things are less hectic, that they are taking days off. We need to get better at providing programs for stress that are easily accessible for all of us and well publicized. (ANM) (White, 2021) p.1532 | U |

Amount of unequivocal (U) findings: 8

Amount of credible (C) findings: 44

(Source: Authors own work)
