# Supplementary material for: Health-care leaders’ experiences of the competencies required for crisis management during COVID-19: a systematic review of qualitative studies
Source: Leadersh Health Serv (Bradf Engl). 2023 May 11;36(4):595–610. doi: 10.1108/LHS-10-2022-0104 (PMC10853848; doi:10.1108/LHS-10-2022-0104)
Supplement: Supplementary file 7 [file leadershhealthserv-36-0595-s007.docx]

Supplementary Table 6 Correspondence between study findings and illustrations provided - not supported

| Study | Finding | Illustration | Level of credibility |
| --- | --- | --- | --- |
| White (2021) | Carrying the burden | N/A ^1^ | NS ^2^ |

^1^ N/A = not available

^2^ NS = not supported

(Source: Authors own work)
