# Supplementary material for: Health-care leaders’ experiences of the competencies required for crisis management during COVID-19: a systematic review of qualitative studies
Source: Leadersh Health Serv (Bradf Engl). 2023 May 11;36(4):595–610. doi: 10.1108/LHS-10-2022-0104 (PMC10853848; doi:10.1108/LHS-10-2022-0104)
Supplement: Supplementary file 8 [file leadershhealthserv-36-0595-s008.docx]

Supplementary Table 7. Summary of findings (ConQual) (Munn et al., 2014)

| Systematic review title: Healthcare leaders’ experiences of the competencies required for crisis management during COVID-19: a systematic review of qualitative studies Participants (P): Healthcare leaders  Phenomena of interest (I): Experiences of competencies required for crisis management  Context (Co): Various types of crises in various healthcare settings | | | | | |
| --- | --- | --- | --- | --- | --- |
| Synthesized finding | Studies (n=8) | Type of research | Dependability | Credibility | ConQual score |
| The competence to comprehend the operational environment | Hølge-Hazelton *et al.*, 2021; Jackson & Nowell, 2021; Vázquez-Calatayud *et al.*, 2022 | Qualitative study | Moderate; Moved down 1 level^1^ | Moderate; Moved down 1 level^2^ | Low |
| The competence to stay resilient amidst change | Abu Mansour & Abu Shosha, 2022; Hølge-Hazelton *et al.*, 2021; Jackson & Nowell, 2021; Vázquez-Calatayud *et al.*, 2022; White, 2021 | Qualitative study | Moderate; Moved down 1 level^1^ | Moderate; Moved down 1 level^2^ | Low |
| The competence to adapt to and manage change | Abu Mansour & Abu Shosha, 2022; Hølge-Hazelton *et al.*, 2021; Jackson & Nowell, 2021; Riddell *et al.*, 2022; Roche *et al.*, 2021; Vázquez-Calatayud *et al.*, 2022; White, 2021 | Qualitative study | Moderate; Moved down 1 level^1^ | Moderate; Moved down 1 level^2^ | Low |
| The competence to manage and take care of staff | Abu Mansour & Abu Shosha, 2022; Hølge-Hazelton *et al.*, 2021; Jackson & Nowell, 2021; Losty & Bailey, 2021; Riddell *et al.*, 2022; Roche *et al.*, 2021; Vázquez-Calatayud *et al.*, 2022; White, 2021 | Qualitative study | Moderate; Moved down 1 level^1^ | Moderate; Moved down 1 level^2^ | Low |
| The competence to co-operate and communicate with diverse stakeholders | Abu Mansour & Abu Shosha, 2022; Losty & Bailey, 2021; Riddell *et al.*, 2022; Roche *et al.*, 2021; Vázquez-Calatayud *et al.*, 2022; White 2021 | Qualitative study | Moderate; Moved down 1 level^1^ | Moderate; Moved down 1 level^2^ | Low |

^1^ There was no statement locating the researcher culturally or theoretically, and the influence of the researcher on the research, and of the research on the researcher, was not addressed.

^2^ Mix of unequivocal/credible findings.

Note: The ConQual Score was calculated as follows: initially, all included studies were ranked as high. The dependability score was calculated using five questions from the critical appraisal criteria (questions Q2-Q4, Q6, Q7). The score was downgraded one level if only two or three responses to the five questions were yes, and downgraded two levels if none or one response to the questions was yes. Next, the ConQual score is formed by calculating the level of credibility of each individual finding. The levels of credibility reflect the degree of congruence between a finding and the accompanying illustration. The findings are classified as being unequivocal, credible, or not supported. Findings that include classifications of both unequivocal and credible are downgraded one level and findings that are classified as equivocal, are downgraded two levels (Munn *et al*., 2014). (Source: Authors own work)
