# Supplementary material for: Health-care leaders’ experiences of the competencies required for crisis management during COVID-19: a systematic review of qualitative studies
Source: Leadersh Health Serv (Bradf Engl). 2023 May 11;36(4):595–610. doi: 10.1108/LHS-10-2022-0104 (PMC10853848; doi:10.1108/LHS-10-2022-0104)
Supplement: Supplementary file 9 [file leadershhealthserv-36-0595-s009.docx]

Supplementary Table 8 Synthesized findings

| Findings (Themes and subthemes) | Categories | Synthesized findings |
| --- | --- | --- |
| Patients transitions through the healthcare system C (Jackson & Nowell, 2021) p.2396 | Ensuring patient-oriented care. The competence to take patients' needs and experiences into consideration. | The competence to comprehend the operational environment. The contradiction between the internal and external operational environment requires skills to understand and acknowledge the various needs of stakeholders while simultaneously ensuring patient-oriented care. |
| Preservation of humanized care C (Vázquez-Calatayud *et al.*, 2022) p.8 |  |  |
| Staff needs versus overall needs C (Hølge-Hazelton *et al.*, 2021) pp.1406-1407 | Balancing the various needs of stakeholders. The competence to recognize the incoherence and complexity of the operational environment. |  |
| Loyalty to own leaders versus staff need C (Hølge-Hazelton *et al.*, 2021) p.1407 |  |  |
| Upholding guidelines versus relatives and patients’ needs C (Hølge-Hazelton *et al.*, 2021) p.1407 |  |  |
| Navigating the political climate C (Jackson & Nowell, 2021) p.2395 |  |  |
| Practicing distance leadership C (Hølge-Hazelton *et al.*, 2021) p.1405 | Learning and transitioning through change. The competence to grow, adapt, reflect, and learn from change. | The competence to stay resilient amidst change. Resilience requires the capability to lead during a period of uncertainty, being able to cope on a personal and professional level, and utilizing skills that one has already acquired through formal education and reflection. |
| Reflections on learning U (White, 2021) p.1531 |  |  |
| Being on a steep learning curve U (Hølge-Hazelton *et al.*, 2021) p.1408 |  |  |
| Management education gives valuable tools U (Hølge-Hazelton *et al.*, 2021) p.1408 |  |  |
| Changing roles and responsibilities C (Jackson & Nowell, 2021) p.2396 |  |  |
| Personal coping C (White, 2021) p.1531 |  |  |
| Personal-development - rewound C (Hølge-Hazelton *et al.*, 2021) p.1408 |  |  |
| Self-awareness U (Abu Mansour & Abu Shosha, 2022) p.389 |  |  |
| Managing uncertainty C (Vázquez-Calatayud *et al.*, 2022) p.84 | Enduring uncertainty throughout change. The competence to stay resilient during uncertainty. |  |
| Planning during uncertainty C (Jackson & Nowell, 2021) p.2395 |  |  |
| Extraordinarily demanding system C (Abu Mansour & Abu Shosha, 2022) p.386 |  |  |
| Physically demanding situations U (Abu Mansour & Abu Shosha, 2022) p.387 |  |  |
| Flexible work approach and practices C (Riddell *et al.*, 2022) pp.8-9 | Embracing new and flexible ways of working. The competence to tolerate change and respond in a creative manner. | The competence to adapt to and manage change. Adaptability in managing change requires the skills to find new solutions which deal with upcoming challenges and situations. |
| Expanded ways of working C (Riddell *et al.*, 2022) p.12 |  |  |
| Removal of organizational barriers C (Riddell *et al.*, 2022) p.7 |  |  |
| Urgent and constant reorganization of the service C (Vázquez-Calatayud *et al.*, 2022) p.83 |  |  |
| Compensating for shortage of materials and human resources C (Abu Mansour & Abu Shosha, 2022) p.388 |  |  |
| Innovation C (Roche *et al.*, 2021) p.4 |  |  |
| Revamping my approach C (White, 2021) p.1530 |  |  |
| Leadership, management and planning C (Roche *et al.*, 2021) p.4 |  |  |
| Complexity of staff management in a changing situation C (Vázquez-Calatayud *et al.*, 2022) p.83 | Coping with changing resources. The competence to be resourceful while managing staff through prioritization and allocation. |  |
| Designating and transferring staff U (Hølge-Hazelton *et al.*, 2021) p.1405 |  |  |
| Workplace transitions in response to COVID-19 C (Jackson & Nowell, 2021) pp.2396-2397 |  |  |
| Maintaining quality through problem-solving C (Jackson & Nowell, 2021) p.2397 |  |  |
| Having a leadership presence C (Losty & Bailey, 2021) p.121 | Maintaining an active grip and presence in leadership. The competence to support staff, participate in decision-making, share awareness of the situation, and maintain open dialog. | The competence to manage and take care of staff. Managing and supporting staff requires skills in managing through knowledge, utilizing the expertise of the staff, and to develop this according to topical needs. |
| Reliance on me U (White, 2021) p.1530 |  |  |
| Maintaining presence, own leadership virtues and professionalism C (Hølge-Hazelton *et al.*, 2021) pp.1404-1405 |  |  |
| Bottom-up decision-making C (Hølge-Hazelton *et al.*, 2021) pp.1407-1408 |  |  |
| Participation in decision-making C (Vázquez-Calatayud *et al.*, 2022) p.84 |  |  |
| Expanded working relationships C (Riddell *et al.*, 2022) pp.6-7 |  |  |
| Knowledge development and dissemination C (Riddell *et al.*, 2022) p.9 |  |  |
| Staff development C (Abu Mansour & Abu Shosha, 2022) p.388 |  |  |
| Maturity of management skills C (Abu Mansour & Abu Shosha, 2022) p.389 |  |  |
| Workforce development and training C (Roche *et al.*, 2021) p.4 | Understanding the capability of the workforce. The competence to identify developmental needs and acknowledge accomplishments. |  |
| Mental toughness C (Losty & Bailey, 2021) pp.121-122 |  |  |
| Prioritization of the biopsychosocial well-being of staff C (Vázquez-Calatayud *et al.*, 2022) pp.84-85 | Taking care of the well-being of staff. The competence to recognize the individuality of the workforce. |  |
| Work that needs attention going forward U (White, 2021) p.1532 |  |  |
| A different kind of support C (White, 2021) p.1530 |  |  |
| Communication is paramount C (Losty & Bailey, 2021) pp.120-121 | Ensuring effective communication. The competence to handle, receive and distribute information at large. | The competence to co-operate and communicate with diverse stakeholders. Co-operation and communication requires skills in multidisciplinary collaboration, being aware of the situation at hand, and being able to convey the necessary information to all parties concerned. |
| Extensive information and communication C (Riddell *et al.*, 2022) p.6 |  |  |
| Communication C (Roche *et al.*, 2021) p.4 |  |  |
| Teamwork C (Vázquez-Calatayud *et al.*, 2022) p.86 | Sustaining teamwork and collaboration. The competence to collaborate, work within a team, give and receive support. |  |
| Professional support C (White, 2021) p.1531 |  |  |
| Collaboration C (Vázquez-Calatayud *et al.*, 2022) p.86 |  |  |
| Colleagues’ support C (Abu Mansour & Abu Shosha, 2022) p.389 |  |  |

U=unequivocal, C=credible (Source: Authors own work)
